# Essential developmental processes in *Physcomitrium patens* require distinct levels of total activity provided by functionally redundant PpROP GTPases

**DOI:** 10.1111/nph.70603

**Published:** 2025-10-05

**Authors:** Aude Le Bail, Benedikt Kost, Janina Nüssel, Tamara Isabeau Lolis, David Koch, Hildegard Voll, Sylwia Schulmeister, Alexander Kaier, Karin Ljung, Maria Ntefidou

**Affiliations:** ^1^ Cell Biology, Department of Biology Friedrich‐Alexander‐University Erlangen‐Nürnberg 91058 Erlangen Germany; ^2^ Biochemistry, Department of Biology Friedrich‐Alexander‐University Erlangen‐Nürnberg 91058 Erlangen Germany; ^3^ Umeå Plant Science Centre, Department of Forest Genetics and Plant Physiology Swedish University of Agricultural Sciences 90183 Umeå Sweden

**Keywords:** apical initial cells, caulonema differentiation, gametophore development, GTP/GDP cycling, *Physcomitrium patens*, plant evolution, polarity, RHO/ROP GTPases

## Abstract

- RHO (RAS homologous) GTPases regulate important cellular and developmental processes in most eukaryotes. Plant‐specific ROP (RHO of plants) GTPase families expanded and functionally diversified during the evolution of vascular plants, but contain few members in nonvascular extant relatives of early land plants. Here, a systematic investigation of essential PpROP functions in the development of the nonvascular moss *Physcomitrium patens* is presented.
- This investigation was based on: knocking out individually or all possible combinations of each of the four Pp*ROP* genes, which encode nearly identical proteins; complementing knockout lines with wild‐type (WT) or mutated PpROPs, or with heterologous homologs; and inducing PpROP overexpression.
- PpROPs were found to have previously unknown functions in cell proliferation, caulonema differentiation, and gametophore formation. PpROP functions were observed to display variable dependence on guanosine diphosphate (GDP)/guanosine triphosphate (GTP) cycling and to rely on distinct downstream signaling. Different cellular and developmental processes were determined to require distinct levels of total PpROP activity, rather than individual PpROPs.
- These observations provide important insights into PpROP functions and signaling in *P. patens*, enhancing our understanding of the evolution of the regulation of developmental processes by ROP/RHO GTPases. The evolutionary origin of the remarkable functional integration and sequence conservation within the PpROP family is discussed.

## Introduction

RHO (RAS homologous) family small guanosine triphosphate hydrolases (GTPases) are expressed in most eukaryotic organisms (Boureux *et al*., 2007) and play key roles in the regulation of essential cellular processes with important functions in the development of multicellular tissues and organs (Etienne‐Manneville & Hall, 2002; Kawano *et al*., 2014). Most RHO GTPases are associated with the plasma membrane (PM) based on posttranslational prenylation, interact with different effectors to trigger downstream signaling specifically in the GTP‐bound conformation, and are inactive when bound to GDP after GTP hydrolysis. While regulatory factors that control RHO activity by promoting GDP for GTP exchange, by stimulating GTP hydrolysis, or by modulating PM association are closely related in different organisms (Berken *et al*., 2005; Kost, 2010; Hodge & Ridley, 2016), RHO‐dependent downstream signaling appears to be much less conserved. Similar cellular processes in animals and in plants are controlled by different families of RHO effectors (Heasman & Ridley, 2008; Yalovsky *et al*., 2008; Müller, 2023; Ntefidou *et al*., 2023). Furthermore, RHO effectors playing key roles in the regulation of important cellular processes in complex vascular plants appear to be either entirely missing (Eklund *et al*., 2010a; Ou & Yi, 2022) or have completely different functions (Ntefidou *et al*., 2023) in nonvascular plants with ancient features.

RHO functions in the control of developmentally relevant cellular processes, including polarization, directional growth, motility, and division, have been extensively characterized both in animals and in plants. However, the direct demonstration of RHO functions in tissue and organ development based on the investigation of loss‐of‐function mutants has been hampered by substantial redundancy within large families of RHO proteins typically expressed in complex multicellular organisms and, possibly, by essential roles of at least some of these proteins in the development of such organisms (Mulvey & Dolan, 2023a).

ROP (RHO of Plants) functions have been most extensively studied in *Arabidopsis thaliana*, a complex flowering plant with an elaborate vascular system, which expresses 11 AtROPs sharing 80–98% amino acid sequence identity (Li *et al*., 1998; Winge *et al*., 2000). The functional characterization of these proteins largely depended on the investigation of loss‐of‐function mutants, and of the effects of overexpressing either wild‐type (WT) AtROPs or mutant versions of these proteins locked in the GTP‐bound conformation (constitutively active) or displaying reduced nucleotide affinity (moderate reduction: fast‐cycling, strong reduction: dominant negative). Different AtROPs were determined to play distinct but partially overlapping (Feiguelman *et al*., 2018) essential roles in the control of the following cellular processes: extremely polarized tip growth displayed by pollen tubes (Li *et al*., 1999; Luo *et al*., 2017) and root hairs (Molendijk *et al*., 2001; Denninger *et al*., 2019); diffuse directional expansion of leaf epidermal pavement cells (Fu *et al*., 2005; Lauster *et al*., 2022); morphogenesis of single‐celled trichomes (Liu *et al*., 2023); and mitotic cell plate positioning required for tissue patterning in developing roots (Roszak *et al*., 2021).

Interestingly, Arabidopsis mutants disrupted in the expression of multiple At*ROP* genes show severely aberrant root hair and/or epidermal leaf pavement cell morphogenesis, but no substantial defects in tissue organization and organ development (Fu *et al*., 2005; Ren *et al*., 2016). Similarly, the development of the liverwort *Marchantia polymorpha* is not strongly affected by knocking out the single Mp*ROP* gene identified in this nonvascular plant, even though defects in the formation of selected tissues and organs were observed (Mulvey & Dolan, 2023a). The relatively weak phenotype of *M*. *polymorpha* knockout mutants lacking MpROP activity obviously cannot be attributed to genetic redundancy. ROP signaling therefore only appears to play a minor role in controlling the development in this liverwort, which displays ancient features presumably similar to those of the extinct common ancestor of all land plants. By contrast, the growth and development of the nonvascular moss *Physcomitrium patens* are severely disrupted in knockout or knock‐down mutants completely lacking or displaying minimal PpROP activity (Burkart *et al*., 2015; Cheng *et al*., 2020; Yi & Goshima, 2020; Bao *et al*., 2022). These mutants only form tiny colonies of irregularly shaped cells, which are unable to directionally expand, differentiate, or form organized tissues. The phenotype of these mutants established that *P. patens* development strictly depends on the activity of the four nearly identical PpROPs expressed in this moss, which share 99–100% amino acid identity and display sequence variability only at positions 148 and 149 outside of known functional domains (Eklund *et al*., 2010a). However, currently available data concerning PpROP functions leave several important questions unanswered: (1) does PpROP activity only exert previously identified functions in the control of cell polarization, directional cell expansion, and mitotic cell plate positioning, or is this activity also required for other cellular and developmental processes? (2) Are different processes controlled by individual ROP isoforms or by the combined activity of multiple PpROPs? (3) Are the four PpROPs functionally redundant or do they differentially contribute to total PpROP activity? (4) Is GDP/GTP cycling required for all identified PpROP functions? (5) Do these functions rely on the same or on distinct downstream signaling? Finally, (6) which evolutionary mechanisms may account for the origin and maintenance of the PpROP protein family with its exceptionally high amino acid sequence conservation?

The development of haploid *P. patens* gametophytes, which are composed of protonemal filaments and gametophores, is an ideal system to further characterize PpROP functions. This process dominates the *P. patens* life cycle, relies on cell polarization, tip growth, strictly controlled cell plate positioning, and additional cellular or developmental processes, and is highly amenable to genetic manipulation based on homologous recombination and clustered regularly interspaced short palindromic repeats/CRISPR‐associated nuclease 9 (CRISPR/Cas9) (Schaefer & Zrÿd, 1997; Cove, 2005; Collonnier *et al*., 2017; Rensing *et al*., 2020). Gametophyte development starts with the germination of haploid spores, which results in the formation of branched filamentous protonemata. These structures can also be induced to develop *in vitro* from isolated protonemal protoplasts or explants, with protoplast‐derived colonies more closely recapitulating normal development (Grimsley *et al*., 1977; Kofuji & Hasebe, 2014). A single apical initial cell at the tip of each protonemal filament expands by tip growth and regularly divides transversely, resulting in filament elongation. The association of different PpROPs with the PM of apical initial cells specifically at the tip, as well as underneath the transversal cell wall separating these cells from subapical cells, is consistent with essential functions of PpROP activity in tip growth and cell plate positioning (Le Bail *et al*., 2019; Cheng *et al*., 2020; Yi & Goshima, 2020). While young protonemal filaments display chloronemal characteristics, the initial cells at their tips undergo gradual caulonema differentiation, which is associated with an increased rate of tip growth and proliferation, a reduction in chloroplast size and number, and the formation of oblique rather than perpendicular transversal cell walls (Jaeger & Moody, 2021). Interestingly, caulonema differentiation is stimulated by the phytohormone auxin (Jang & Dolan, 2011) and has recently been demonstrated to be effectively suppressed by the PpROP effector PpRIC downstream of auxin‐induced changes in gene expression (Ntefidou *et al*., 2023). Filament branching as well as the formation of lateral buds developing into gametophores are initiated by the asymmetric division of subapical caulonemal cells. As this process is defective in mutants lacking three of the four PpROPs, it also appears to be controlled by PpROP activity (Yi & Goshima, 2020). Mature gametophores are composed of leafy shoots with leaf‐like phyllids and filamentous rhizoids, which are free of chloroplasts but otherwise closely resemble caulonemal filaments and elongate based on the same mechanisms. Reproductive organs produced at the tip of mature gametophores mediate sexual reproduction, which initiates sporophyte development corresponding to the short diploid phase of the life cycle.

Open questions regarding PpROP functions during *P. patens* gametophyte development were addressed through comprehensive knockout (disruption of all Pp*ROP* genes individually and in all possible combinations), complementation, and overexpression experiments, combined with quantitative characterization of resulting phenotypes. These investigations revealed that, beyond their previously established roles in cell polarization, mitotic cell plate positioning, and directional cell expansion, PpROPs also have essential functions in controlling caulonema differentiation, gametophore formation, and cell proliferation. Data obtained further demonstrate that different PpROP functions vary in their dependence on GDP/GTP cycling and require distinct downstream signaling. Additionally, these data firmly establish that the four PpROPs are functionally redundant, as was previously hypothesized based on their high amino acid sequence conservation and on the phenotypic characterization of selected knockout mutants (Burkart *et al*., 2015; Cheng *et al*., 2020; Yi & Goshima, 2020; Bao *et al*., 2022). Importantly, comprehensive investigation of all possible knockout phenotypes and of the complementation of quadruple knockout mutants by PpROP2 expressed at variable levels under the control of an estradiol‐titratable promoter revealed that different cellular and developmental processes require distinct levels of total PpROP activity rather than individual PpROPs. Together, these findings demonstrate that the exceptional sequence conservation within the PpROP gene family is accompanied by remarkable functional integration. Evolutionary mechanisms potentially underlying the origin and maintenance of the PpROP gene family with its unique features are discussed, laying the groundwork for more comprehensive future investigations into this intriguing question.

## Materials and Methods

### Plant materials and culture conditions

*Physcomitrium patens* ecotype Gransden (Hedw.) Mitt., WT strain (2012; Ashton & Cove, 1977) and transgenic lines (Supporting Information Table S1) were grown axenically at 25°C under continuous white light illumination with fluorescent tubes (Philips Master TL‐D Super 80 58 W, cool white) at an intensity of 50 μmol m^−2^ s^−1^. Unless otherwise stated, moss protonemata were cultivated and analyzed in 9‐cm Petri dishes on BCDA medium (Cove *et al*. 2009) (1 mM MgSO_4_, 1.84 mM KH_2_PO_4_, 10 mM KNO_3_, 5 mM ammonium‐tartrate, 1 mM CaCl_2_, 45 μM FeSO_4_, 9.9 μM H_3_BO_3_, 2 μM MnCl_2_, 116 nM AlK[SO_4_]_2_, 424 nM CoCl_2_, 220 nM CuSO_4_, 235 nM KBr, 168 nM KI, 660 nM LiCl, 124 nM SnCl_2_, and 191 nM ZnSO_4_; Ashton & Cove, 1977) solidified with 0.7% (w/v) agar and supplemented with 100 μg ml^−1^ vancomycin (Duchefa Biochemie) unless other antibiotics were used for transgene selection. Solidified medium was covered with cellophane disks (AA Packaging; Grimsley *et al*., 1977) except for long‐term cultivation of mutant moss cultures consisting of small clumps of round cells. Protonemata were sub‐cultured every 7 d and regenerated every 6 wk from gametophores by homogenization (OMNI International) in Milli‐Q H_2_O (Merck, Darmstadt, Germany). Gametophore‐forming colonies were cultivated and analyzed on BCD medium (BCDA without ammonium‐tartrate, supplemented with 100 μg ml^−^^1^ vancomycin) solidified with 0.7% (w/v) agar or in liquid BCD medium. Titratable expression of RHO GTPases was induced by adding β‐estradiol (Sigma), dissolved in dimethyl sulfoxide at final concentrations from 0.02 nM to 1 μM to BCDA or BCD medium after autoclaving. The culture conditions used in each experiment are listed in Table S2.

### Generation of mutants, complemented mutants, and overexpression lines

To generate *rop* knockout (KO) mutants, homologous recombination (Schaefer *et al*., 1991) was employed to replace all Pp*ROP* genes individually and in every possible combination by antibiotic resistance genes (Figs S1a, S2a,c, S3a). Knockout lines obtained were verified by PCR genotyping (Figs S1a,b, S2, S3). Higher‐order mutants were generated sequentially, starting with *rop1*, *rop2*, *rop3*, and *rop4* single knockout lines (Fig. S1c: upper panel). At least two independent lines per genotype were generally characterized, except for the *rop2*, *rop4*, r*op4/1*, and *rop2/4/1* genotypes, which were only obtained once. Two independent *rop* ^4xKO^ lines were generated by knocking out either Pp*ROP1* in the *rop3/2/4* background (*rop3/2/4/1*) or Pp*ROP2* in the *rop3/4/1* background (*rop3/4/1/2*; Fig. S1c: upper panel). Independently generated mutants lacking functional copies of the same Pp*ROP* genes consistently displayed indistinguishable phenotypes.

Different *rop* ^4xKO^/*ROP1* ^pro^:*ROPX* lines expressing either PpROP1, PpROP2, or PpROP3 under the control of the Pp*ROP1* promoter in an identical *rop* ^4xKO^ background were generated based on homologous recombination. The coding sequence (exons and introns) and 3′ untranslated region (UTR) of the endogenous Pp*ROP1* gene in the *rop3/2/4* background was replaced by coding sequence (CDS) fragments encoding PpROP1, PpROP2, or PpROP3, which were attached to the Pp*ROP1*, Pp*ROP2*, or Pp*ROP3* 3′ UTR, respectively (Fig. S1a,c: lower panel). Resulting *rop* ^4xKO^/*ROP1* ^pro^:*ROPX* lines (at least two per genotype) were confirmed by PCR genotyping (Fig. S1b). In all cases, the investigation of independently generated genotypically identical lines produced indistinguishable results.

Using the strategy outlined in the previous paragraph, the coding sequence (exons and introns) and 3′ UTR of the endogenous Pp*ROP1* gene in the *rop3/2/4* background was also replaced by CDS fragments encoding GTP‐locked PpROP1^Q64L^ or fast‐cycling PpROP1^F31L^, which were attached to the Pp*ROP1* 3′ UTR (Fig. S1a,c: lower panel). Individual *rop* ^4xKO^/*ROP1* ^pro^:*ROP1* ^Q64L^ and *rop* ^4xKO^/*ROP1* ^pro^:*ROP1* ^F31L^ lines expressing mutated Pp*ROP1* genes at the same level as endogenous Pp*ROP1* expression were confirmed by PCR genotyping (Fig. S1b) and phenotypically characterized.

Homologous recombination was also employed to generate two independent *rop* ^4xKO^/*ind* ^pro^:*ROP2* lines, which expressed PpROP2 under the control of an estradiol‐inducible LexA/35S promoter (Kubo *et al*., 2013) in a *rop* ^4xKO^ background. The coding sequence (exons and introns) and 3′ UTR of the Pp*ROP3* gene in the *rop2/4/1* background were replaced by a CDS fragment encoding PpROP2, which was fused at 5′‐end to the estradiol‐inducible LexA/35S promoter and at the other end to the Pp*ROP2* 3′ UTR (Figs S1c: lower panel, S2c). Two *rop* ^4xKO^/*ind* ^pro^:*ROP2* lines were obtained, which were confirmed by PCR genotyping (Fig. S2d) and phenotypically indistinguishable under the conditions investigated.

To generate WT/*ind* ^pro^:*ROP1* lines exhibiting estradiol‐inducible PpROP1 overexpression in the WT background, a CDS fragment encoding PpROP1, which was fused at the 5′‐end to the estradiol‐inducible LexA/35S promoter (Kubo *et al*., 2013) and at the other end to the Pp*ROP1* 3′ UTR, was introduced into a neutral region (Schaefer *et al*., 1991) of the WT genome based on homologous recombination (Fig. S1d). One of the WT/*ind* ^pro^:*ROP1* lines obtained, which displayed massive estradiol‐inducible Pp*ROP1* overexpression, was confirmed by PCR genotyping (Fig. S1e) and selected for phenotypic characterization.

Multiple *rop* ^4xKO^/*ind* ^pro^:At*ROP7* and *rop* ^4xKO^/*ind* ^pro^:Hs*RHOA* lines, which expressed AtROP7 or HsRHOA under the control of the estradiol‐inducible LexA/35S promoter (Kubo *et al*., 2013) in the same *rop* ^4xKO^ background, were established based on homologous recombination. To this end, the coding sequence (exons and introns) of the endogenous Pp*ROP4* gene in the *rop3/1/2* background was replaced by CDS fragments encoding AtROP7 or HsRHOA, which were fused at 5′‐end to the estradiol‐inducible LexA/35S promoter (Kubo *et al*., 2013; Figs S1c: lower panel, S3a). Lines displaying maximal At*ROP7* or Hs*RHOA* transcript levels upon estradiol induction were confirmed by PCR genotyping (Fig. S3b) and selected for phenotypic characterization.

### Phenotypic analysis of protonemata regenerated from protoplasts

Protonemata regenerated from protoplasts were prepared as described previously (Le Bail *et al*., 2019) based on Cove *et al*. (2009). In brief, 4‐ to 7‐d‐old protonemata were digested with 0.5% (w/v) driselase (Sigma) in 8.5% mannitol and regenerated on PRMB medium (supplemented with 100 μg ml^−1^ vancomycin) solidified with 0.7% agar in Petri dishes covered with cellophane for 2 d, followed by transfer of the cellophane disks to BCDA medium (supplemented with 100 μg ml^−1^ vancomycin). BCDA medium was treated with 0.02 nM^−1^ μM β‐estradiol for Pp*ROP1* overexpression (WT/*ind* ^pro^:*ROP1*) or complementation of *rop* ^4xKO^ with an inducible *ROP* or *RHO* expression construct (*rop* ^4xKO^/*ind* ^pro^:ROPX or *rop* ^4xKO^/*ind* ^pro^:*RHO*). Nuclei were counted 4 d after protoplast preparation. Cell length, caulonema differentiation, and protonemal area were measured at 5 d. To analyze the gametophore stage, individual protonemal colonies, having regenerated for 5 d on BCDA, were transferred to BCD medium (supplemented with 100 μg ml^−1^ vancomycin) without cellophane, and the colony area was measured 4 or 5 wk after protoplast preparation. The culture conditions used in each experiment are listed in Table S2. Experiments were repeated three times. The cell length and the area of 5‐d‐old and 5‐wk‐old colonies of *rop* ^1xKO^, *rop* ^*2x*KO^, and *rop* ^3xKO^ lines were analyzed simultaneously.

### Bright‐field microscopy of cell length, caulonema differentiation, and colony size

To determine the cell length and caulonema differentiation, filaments with three or more cells of 5‐d‐old protonemata regenerated from protoplasts were imaged using a wide‐field microscope (DMI4000B; Leica, Wetzlar, Germany). The length of subapical cells was analyzed using the Fiji software (ImageJ 1.54f; Schindelin *et al*., 2012). Caulonema differentiation, a gradual process that occurs over two or more cell divisions (Jang & Dolan, 2011) and often results in the formation of intermediate cell types along filaments, was assessed by optical inspection of protonemal filaments and comparing the cellular characteristics of the apical cell to those of the basal cell, which is consistently chloronemal. Filaments were classified as caulonemata if the apical cell met all of the following criteria: (1) presence of small and few chloroplasts; (2) oblique cross walls; and (3) elongated and narrower cell morphology. Only filaments whose apical cells exhibited all three characteristics were scored as caulonemata. The percentage of caulonema differentiation was calculated as the ratio of caulonemal filaments to the total number of filaments observed. The size of 5‐d‐old protonemata was determined based on Chl autofluorescence (excitation: 450–490 nm; emission: 500 nm long‐pass) images obtained with a fluorescence stereo microscope (M205 FA; Leica) and analyzed using a Fiji macro (Vidali *et al*., 2007). Four‐ to five‐week‐old colonies were imaged using the stereo microscope (M205 FA; Leica), and their size was determined by applying a thresholding to convert reflected light 8‐bit images to 1‐bit images, which were analyzed using the Fiji software (ImageJ 1.54f; Schindelin *et al*., 2012).

### Generation of knockout constructs

Molecular cloning was performed with standard methods (Green & Sambrook, 2012) using Phusion DNA polymerase (Thermo Fisher Scientific, Waltham, MA, USA), the primers listed in Table S3 (Eurofins Genomics, Ebersberg, Germany), restriction enzymes, and T4 Ligase (New England Biolabs, Ipswich, MA, USA). The resulting constructs (Table S4) were verified by Sanger sequencing (Eurofins Genomics). To knock out Pp*ROP*s (*rop1*, *rop2*, *rop3*, and *rop4*) based on homologous recombination, genomic fragments of 0.7–1.0 kb upstream of the start codon (5′ target) and downstream of the stop codon (3′ target) were cloned flanking a resistance marker. The backbone of pMT123 (Thelander *et al*., 2004) was used for cloning into the XhoI/HindIII and SpeI/NotI sites, resulting in pSLU33 for knockout of Pp*ROP1* (Fig. S1a,b); the p35S‐Zeo backbone (Hiwatashi *et al*., 2008) was used for cloning pSLU34 for knockout of Pp*ROP2* (Fig. S2a,b); the p35S‐loxP‐BSD backbone (Li *et al*., 2017) was used for cloning pSLU35 for knockout of Pp*ROP3* (Fig. S2c,d); and the pMT164 backbone (Thelander *et al*., 2007) was used for cloning pSLU36 for knockout of Pp*ROP4* (Fig. S3).

### Generation of expression constructs

To complement *rop* ^4xKO^ with Pp*ROP1* ^Q64L^ or Pp*ROP1* ^F31L^ expressed by the endogenous Pp*ROP1* promoter, the Pp*ROP1* coding and 3′ UTR sequence were amplified from cDNA and cloned into pENTR™/D‐Topo (Thermo Fisher Scientific), yielding pFAU253 and mutated using PCR‐based site‐directed mutagenesis followed by DpnI digestion, generating pFAU256 (Pp*ROP1* ^Q64L^) or pFAU417 (Pp*ROP1* ^F31L^). The Pp*ROP1* ^Q64L^ or Pp*ROP1* ^F31L^ fragments were transferred into the SalI/HindIII sites of pMT123 (Thelander *et al*., 2004) and flanked by 0.7‐ to 1.0‐kb regions upstream of the start codon (5′ target) or downstream of the stop codon (3′ target) of Pp*ROP1*, enabling the replacement of the Pp*ROP1* locus in *rop3/2/4* through homologous recombination, yielding pFAU306 (*rop* ^4xKO^/*ROP1* ^pro^:*ROP1* ^Q64L^) or pFAU421 (*rop* ^4xKO^/*ROP1* ^pro^:*ROP1* ^F31L^; Fig. S1a,b; Table S4).

To complement *rop* ^4xKO^ with a single Pp*ROP* under the control of the Pp*ROP1* promoter, the full‐length coding sequences of Pp*ROP2* or Pp*ROP3*, including the respective 3′ UTR, were amplified from cDNA and cloned into pENTR™/D‐Topo, generating pFAU269 (Pp*ROP2*) and pFAU270 (Pp*ROP3*). Each Pp*ROP* fragment was then transferred from pFAU253, pFAU269, or pFAU270 into the SalI/HindIII sites of pFAU306, which enabled the replacement of the Pp*ROP1* locus in *rop3/2/4* through homologous recombination, yielding pFAU508 (*rop* ^4xKO^/*ROP1* ^pro^:*ROP1*), pFAU509 (*rop* ^4xKO^/*ROP1* ^pro^:*ROP2*), and pFAU517 (*rop* ^4xKO^/*ROP1* ^pro^:*ROP3*), respectively (Fig. S1a,b).

To complement *rop* ^4xKO^ with a titratable expression of a *ROP* or *RHO GTPase*, the LexA operator and the XVE chimeric sequence were amplified by PCR from pGX8 (Kubo *et al*., 2013) and cloned into the NotI/EcoRV sites of pSLU35, yielding pFAU425, and into the SpeI/PmeI/PmlI sites of pSLU36, yielding pFAU391. The coding and 3′ UTR sequence of Pp*ROP2* were transferred from pFAU269 to pFAU425 by Gateway LR reaction (Thermo Fisher Scientific; Katzen, 2007), generating pFAU431 (*rop* ^4xKO^/*ind* ^pro^:*ROP2*; Fig. S2c,d) which was used for replacement of the Pp*ROP3* locus based on homologous recombination in *rop2/4/1*. The full‐length At*ROP7* and Hs*RHOA* coding sequences were amplified from *Arabidopsis thaliana* or *Homo sapiens* cDNA, respectively, and cloned into pENTR/D‐Topo (Thermo Fisher Scientific), yielding pFAU290 (At*ROP7*) and pFAU294 (Hs*RHOA*), followed by Gateway LR reaction (Thermo Fisher Scientific; Katzen, 2007) with pFAU391, generating pFAU398 (*rop* ^4xKO^/*ind* ^pro^:At*ROP7*) or pFAU402 (*rop* ^4xKO^/*ind* ^pro^:Hs*RHOA*) for gene replacement based on homologous recombination of the Pp*ROP4* locus in *rop3/1/2* (Fig. S3).

To overexpress Pp*ROP1* (WT/*ind* ^pro^:*ROP1*) from the β‐estradiol‐inducible promoter, the coding sequence of Pp*ROP1*, including the 3′ UTR, was transferred from pFAU253 by Gateway LR reaction (Thermo Fisher Scientific; Katzen, 2007) into pGX8 (Kubo *et al*., 2013), generating pFAU461 (Fig. S1d) used for transgene integration based on homologous recombination into the neutral genomic region of PIG1b (Okano *et al*., 2009).

To express Pp*PINA*‐*eGFP* under the endogenous Pp*PINA* promoter, the pFAUobt63 vector was obtained from Mattias Thelander (Viaene *et al*., 2014) for the knock‐in of eGFP into the PINA locus of *P. patens* WT and *rop1/2* double knockout.

### *Physcomitrium patens* transformation

Gene targeting into the *P. patens* genome by homologous recombination was previously described for polyethylene glycol (PEG)‐mediated transformation (Le Bail *et al*., 2019) based on the method by Schaefer & Zrÿd (1997). In brief, 1.6 × 10^6^ protoplasts were transformed with 15 μg of linearized vector and allowed to regenerate for 5 d on PRMB medium, followed by two rounds of selection alternating between BCDA medium containing the appropriate antibiotic (30 μg ml^−1^ hygromycin B (Carl Roth), 20 μg ml^−1^ G418 (Merck), 50 μg ml^−1^ zeocin (Thermo Fisher Scientific), or 75 μg ml^−1^ blasticidin (InvivoGen)) according to the transformed resistance cassette and BCDA medium (supplemented with 100 μg ml^−1^ vancomycin). Transgenic lines were genotyped through PCR using primers indicated in Table S3 and Figs S1–S3.

### Gene expression analysis by RT‐qPCR

Total RNA was extracted from 1‐wk‐old protonemata cultivated through homogenization or from 7‐ to 10‐d‐old protonemata regenerated from protoplasts. Samples were frozen in liquid nitrogen and lysed with glass beads using TissueLyser II (Qiagen). Total RNA was isolated using a Nucleospin RNA Plus™ Kit (Macherey‐Nagel, Dueren, Germany), and RNA integrity was assessed using agarose gel electrophoresis. First‐strand cDNA was synthesized from 1 μg of total RNA using iScript™ Reverse Transcription Supermix for RT‐qPCR (Bio‐Rad) and diluted 1 : 20 in Milli‐Q H_2_O. Reverse transcription quantitative polymerase chain reaction (RT‐qPCR) was conducted using SscAdvanced™ Universal SYBR Green Supermix (Bio‐Rad) and a CFX96™ thermal cycler (Bio‐Rad). Total RNA was prepared from three moss samples (biological replicates), and each cDNA sample was measured in two RT‐qPCR reactions (technical replicates). Absolute transcript levels were quantified with standard curves prepared from a 10‐fold dilution series of genomic DNA from 1‐wk‐old protonemata using the PhytoPure DNA Extraction Kit (Cytiva, Marlborough, MA, USA). Pp*UBIQUITIN‐E2* served as a reference gene (Le Bail *et al*., 2013). Primers used for RT‐qPCR are listed in Table S3. Experiments were repeated at least two times.

### Quantification of free IAA

Seven‐day‐old protonemata tissues (*c*. 15–30 mg fresh weight per sample) were extracted after the addition of 500 pg ^13^C6‐IAA internal standard per sample, purified, and analyzed for free indole‐3‐acetic acid (IAA) concentration using combined gas chromatography‐tandem mass spectrometry as described (Andersen *et al*., 2008).

### Confocal microscopy, counting of nuclei, and cell wall visualization

A Leica TCS SP8 DIVE‐FALCON inverted confocal laser scanning microscope with an HC PL APO CS2 20X/0.75 NA water immersion lens was used for confocal imaging, operated by the Application Suite X software. PINA‐GFP fluorescence was excited using an argon laser at 488 nm and detected in a 500–550‐nm emission window. To determine cell numbers, nuclei were counted in 4‐d‐old protonemata regenerated from protoplasts, which were grown on solid medium overlaid with a cellophane sheet. Nuclei were stained with 4′,6‐diamidino‐2‐phenylindole (DAPI) according to the method of Vidali *et al*. (2010). In brief, small cellophane sectors covered with protonemata were excised from culture plates and transferred onto glass slides. After 30‐min incubation at room temperature in fixation and staining solution (100 mM PIPES, pH 6.8, 0.1% (v/v) Nonidet P‐40, 2% (w/v) paraformaldehyde, and 0.1 μg ml^−1^ DAPI), protonemata were covered with a cover slip and imaged by recording Z‐stacks of optical sections at 2 μm spacing between the most proximal and distal surfaces. DAPI fluorescence was excited using a DMOD laser at 405 nm and detected in a 430–480 nm emission window. Chl autofluorescence was simultaneously excited using an argon laser at 514 nm and detected in a 525–575‐nm emission window. Entire Z‐stacks were inspected to count nuclei in all optical planes. Cell walls in young protonemata and developing gametophores were labeled with 10 μg ml^−1^ propidium iodide solution (Merck) and imaged by recording Z‐stacks of optical sections at 2 μm spacing. Propidium iodide fluorescence was excited using a DPSS laser at 561 nm and detected in a 580–600‐nm emission window. The Fiji software (Schindelin *et al*., 2012) was employed to generate maximum Z‐stack projections.

### Alignment analysis

ROP homologs in the moss species listed in Table S7 were identified using the full‐length amino acid sequence of PpROP1 as a query. In *Sphagnum fallax*, ROPs were identified with the Protein Basic Alignment Search Tool (Blastp; Altschul *et al*., 1990) using the genome database of *S. fallax* v1.1 (Phytozome genome ID:522; Healey *et al*., 2023) available at the Phytozome (v.13) depository (https://phytozome‐next.jgi.doe.gov) (Goodstein *et al*., 2012). For all other moss species lacking genome data, ROP homologs were identified with Translated Blast (tBlastn) using the 1000 plant (1KP) transcriptomes data depository (https://db.cngb.org/onekp/) (Leebens‐Mack *et al*., 2019). A protein alignment of full‐length ROP sequences and a protein identity matrix were obtained using UniProt (The UniProt Consortium, 2025).

### Statistical analysis

GraphPad Prism (v.10.3.1; GraphPad software, Boston, MA, USA) was used for data analysis. Unpaired Student's *t*‐tests were used to determine significant differences between two genotypes. One‐ or two‐way analysis of variance (ANOVA, Welch's ANOVA) followed by pairwise multiple comparison tests (Tukey's/Dunnett's) was employed for three or more genotypes, as indicated in the figure legends. Significance to WT and comparisons relevant to data interpretation are indicated in graphs (detailed statistical results are given in Table S5).

## Results

### Individual Pp*ROP* genes are not essential for protonemal development

Previously reported RT‐qPCR and RNA‐sequencing data demonstrated similar expression levels of all four Pp*ROP* genes in developing protonemata (Perroud *et al*., 2018, Ntefidou *et al*., 2023). RT‐qPCR analyses performed here essentially confirmed these observations, although Pp*ROP4* was found to display *c*. 2× higher transcript levels as compared to the other three genes (Fig. S4). These data indicate that all four PpROPs may contribute to the control of protonemal growth and/or differentiation.

To systematically investigate PpROP functions in protonemal development, homologous recombination (Schaefer *et al*., 1991) was employed to knock out all Pp*ROP* genes individually and in every possible combination (Figs S1–S3). To enable comparative quantitative characterization of knockout phenotypes, protonemata regenerated from isolated protoplasts were investigated at defined developmental stages. None of the four single *rop* knockout mutants (*rop* ^1xKO^) displayed evident defects in: the length of fully expanded subapical chloronemal or caulonemal cells (Fig. S5a); the morphology of the tips of chloronemal or caulonemal filaments (Fig. S5b); the rate of caulonema differentiation (Fig. S5c), which is enhanced in a knockout mutant lacking the PpROP effector PpRIC (*ric‐1* ^KO^, Ntefidou *et al*., 2023); or the size or morphology of 5‐d‐old protonemata or of 5‐wk‐old colonies (Fig. S5d–f). These observations establish that each individual *ROP* gene is dispensable for cell expansion and differentiation during protonemal development.

### Mutants lacking any combination of two Pp*ROP* genes display enhanced caulonema differentiation

**Fig. 1:**
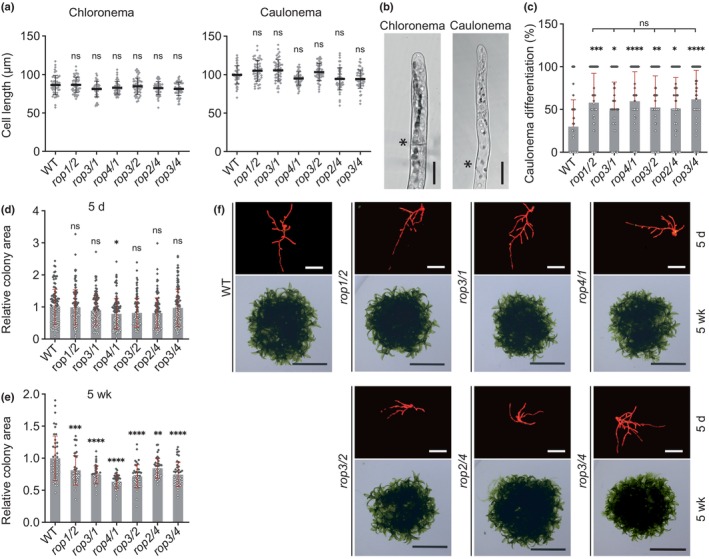
Knockout of two Pp*ROP*s (*rop* ^2xKO^) promotes caulonema differentiation. Graphs and images based on 5‐d‐old *Physcomitrium patens* protonemata or 5‐wk‐old colonies regenerated from protoplasts with the indicated genotypes of double Pp*ROP* knockout mutants and the wild‐type (WT) using culture conditions listed in Supporting Information Table S2. (a) Average subapical cell length of chloronema and caulonema cells in 5‐d‐old protonemata. *n* = 50 cells were analyzed per genotype. The experiment was performed three times with consistent results. (b) Bright‐field images of 5‐d‐old chloronemal and caulonemal filament tips of the WT (the tips of mutant filaments are shown in Fig. S6a). Asterisks indicate the cell wall between neighboring cells. Bars, 25 μm. (c) Average percentage of caulonema differentiation in 5‐d‐old protonemal filaments with at least three cells as determined by microscopic observation. *n* = 60 colonies per genotype were measured in three independent experiments. (d, e) Average size (area) of 5‐d‐old protonemata (d) or 5‐wk‐old colonies (e) as determined based on microscopic imaging of Chl autofluorescence (f, 5‐d‐old) or pictures recorded using a stereo microscope (f, 5‐wk‐old). The mean value of WT was used as a calibrator (relative area = 1). *n* = 105 colonies per genotype were measured in 3 independent experiments (d), or *n* = 42 colonies per genotype were measured. The experiment was performed three times with consistent results (e). Bars, 400 μm (f, 5‐d‐old), 10 mm (f, 5‐wk‐old). (a, c–e) Error bars: SD; dots represent individual data points. Statistical analysis by one‐way ANOVA/Tukey's test (pairwise comparisons to WT and relevant pairwise comparisons are displayed; all others see Table S5): ^ns^, *P* > 0.05 (not significant); *, *P* ≤ 0.05; **, *P* ≤ 0.01; ***, *P* ≤ 0.001; **** *P* ≤ 0.0001.

The six *rop* ^2xKO^ mutants lacking two Pp*ROP* genes in all possible combinations did not show detectable defects in cell expansion or morphology in 5‐d‐old protonemata, except for an apparent minor decrease in the size of *rop4/1* protonemata (Figs 1a,b,d,f, S6a). However, at the same developmental stage, caulonema differentiation was substantially enhanced to a similar extent in all *rop* ^2xKO^ mutants (Fig. 1c), demonstrating an important function of PpROP activity in the suppression of this process. Caulonema differentiation is tightly correlated with enhanced cell expansion, indicating interdependence between these processes (Jang & Dolan, 2011). Since the length of chloronemal cells was not affected in *rop* ^2xKO^ mutants (Fig. 1a), enhanced caulonema differentiation displayed by these mutants is not a secondary effect of altered cell expansion, but appears to be a direct consequence of reduced PpROP activity.

As none of the *rop* ^1xKO^ mutants showed enhanced caulonema differentiation (Fig. S5c), any combination of three Pp*ROP* genes evidently is sufficient for the inhibition of this process in normally developing protonemata. PpROP activity could potentially suppress caulonema differentiation by interfering with auxin signaling, which plays an important role in stimulating this process in *P. patens* (Jang & Dolan, 2011; Jaeger & Moody, 2021). To investigate this possibility, levels of IAA, the primary naturally occurring auxin‐family phytohormone, were analyzed in *rop* ^2xKO^ mutants, alongside the distribution of an auxin‐transport marker and auxin‐dependent gene expression. All *rop* ^2xKO^ mutants were found to: contain WT IAA levels (Fig. S7a); display normal accumulation of the key auxin transporter PpPINA (PIN‐FORMED A auxin efflux carrier; Viaene *et al*., 2014) specifically in the PM at the tip of apical protonemal cells (Fig. S7b); and express at normal levels different auxin‐responsive genes (Fig. S7c). The genes selected for this analysis are either required for auxin‐induced caulonema differentiation (Pp*RSL1*; *ROOTHAIR DEFECTIVE SIX‐LIKE1*; Jang *et al*., 2011), or have essential functions in auxin biosynthesis (Pp*SHI1*; *SHORT INTERNODES1*; Eklund *et al*., 2010b), transport (Pp*PINA*), or signaling (Pp*AUX1*; *AUXIN RESISTANT 1*; Lavy *et al*., 2016). Together, these observations establish that PpROP activity inhibits caulonema differentiation either downstream of, or independently from, auxin‐controlled gene expression.

As pointed out in the Introduction section, caulonemal filaments exhibit significantly higher growth rates than chloronemal filaments (Cove & Knight, 1993). Interestingly, despite enhanced caulonema differentiation and although 5‐d‐old *rop* ^2xKO^ protonemata did not display substantial growth defects (Figs 1a,b,d,f, S6a), the size of 5‐wk‐old *rop* ^2xKO^ colonies was significantly reduced (Fig. 1e,f). Consistent with this observation, PpROP activity not only inhibits caulonema differentiation but also promotes tip growth required for cell and protonemata expansion, as reported previously (Burkart *et al*., 2015; Cheng *et al*., 2020; Yi & Goshima, 2020; Bao *et al*., 2022). However, it is important to note that *rop* ^2xKO^ protonemata displayed minimal growth defects, which only became apparent late during development and did not prevent the demonstration of enhanced caulonema differentiation at an earlier stage.

### Cell and protonemal expansion are severely disrupted in mutants lacking any combination of three Pp*ROP* genes

**Fig. 2:**
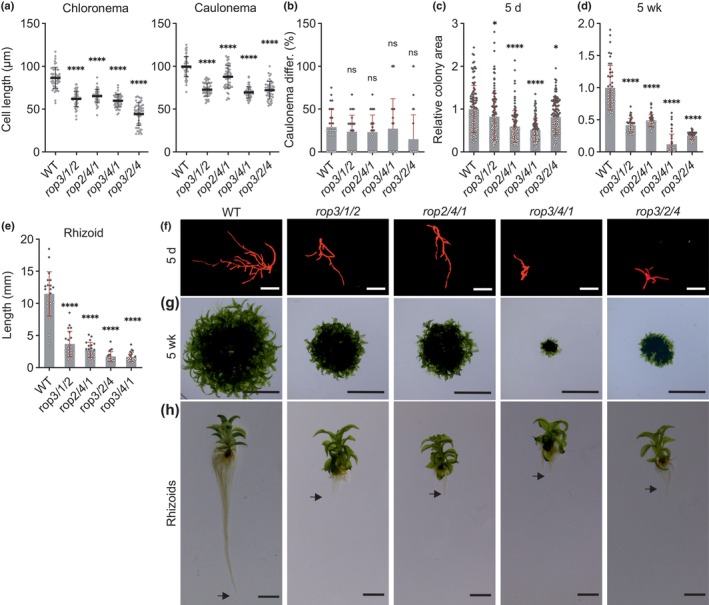
Knockout of three Pp*ROP*s (*rop* ^3xKO^) inhibits tip growth of protonemata and rhizoids. Graphs and images are based on 5‐d‐old *Physcomitrium patens* protonemata or 5‐wk‐old colonies regenerated from protoplasts with the indicated genotypes of triple Pp*ROP* knockout mutants and the wild‐type (WT) cultivated on media listed in Supporting Information Table S2. (a) Average subapical cell length of chloronemal and caulonemal cells in 5‐d‐old protonemata (images of the tips of WT and mutant filaments are displayed in Fig. S6b). *n* = 50 cells per genotype were measured. The experiment was repeated three times with consistent results. (b) Average percentage of caulonema differentiation in 5‐d‐old protonemal filaments with at least three cells as determined by microscopic observation. *n* = 60 colonies per genotype were measured in three independent experiments. (c, d, f, g) Average size (area) of 5‐d‐old protonemata (c) or 5‐wk‐old colonies (d) as determined based on microscopic imaging of Chl autofluorescence (f) or pictures recorded using a stereo microscope (g). Bars: 400 μm (f); 10 mm (g). The mean value of WT was used as a calibrator (relative area = 1). *n* = 105 colonies per genotype were measured in three independent experiments (c), or *n* = 45 colonies per genotype were measured. The experiment was repeated three times with consistent results (d). (e, h) Average length of rhizoids of 5‐wk‐old gametophores (e) based on pictures recorded using a stereo microscope (h). Arrows indicate the end of rhizoids. Bars, 5 mm. *n* = 21 rhizoids per genotype were measured. The experiment was repeated three times with consistent results. (a–e) Error bars: SD; dots represent individual data points. Statistical analysis by one‐way ANOVA/Tukey's test. Comparisons to WT are displayed, all others see Table S5: ^ns^, *P* > 0.05 (not significant); *, *P* ≤ 0.05; ****, *P* ≤ 0.0001.

Consistent with previously reported phenotypic analyses of *P. patens rop1/2/3* and *rop2/3/4* triple knockout mutants (Cheng *et al*., 2020; Yi & Goshima, 2020), 5‐d‐old protonemata of each of the four *rop* ^3xKO^ mutants lacking three Pp*ROP* genes in all possible combinations displayed severe growth defects (Figs 2a,c,f, S6b). Already at this early developmental stage, the length of subapical chloronemal and caulonemal cells (Figs 2a, S6b), as well as the size of protonemata (Fig. 2c,f), was substantially decreased. Interestingly, at the same developmental stage and in contrast to *rop* ^2xKO^ mutants, *rop* ^3xKO^ mutants did not display enhanced caulonema differentiation (Fig. 2b). In accordance with the previously reported tight correlation between caulonema differentiation and enhanced cell expansion (Jang & Dolan, 2011), reduced cell expansion appears to inhibit caulonema differentiation in *rop* ^3xKO^ protonemata.

The analysis of 5‐wk‐old *rop* ^3xKO^ colonies revealed even more pronounced growth defects (Fig. 2d,g). Interestingly, and consistent with previously reported observations (Yi & Goshima, 2020), at this developmental stage, *rop* ^3xKO^ colonies formed gametophores with small but morphologically essentially normal leafy shoots and even more severely stunted rhizoids (Fig. 2e,g,h), which, like protonemata, are composed of filaments that elongate based on apical tip growth. The phenotypic characterization of *rop* ^3xKO^ protonemata and gametophores therefore not only confirms essential functions of PpROP activity in the control of tip growth but also indicates additional roles of this activity in other forms of directional cell expansion, which are required for leafy shoot development. Whereas tip growth is strongly reduced in all *rop* ^3xKO^ mutants (Figs 2, S6b), this process is only marginally affected in all *rop* ^2xKO^ mutants (Figs 1, S6a). The expression of any combination of two PpROPs therefore appears to be sufficient to support nearly normal tip growth.

### All four PpROPs are equally capable of promoting tip growth

Amino acid sequence identities of at least 99% within the PpROP protein family, together with highly similar phenotypes of all *rop* ^2xKO^ (Fig. 1) and *rop* ^3xKO^ (Fig. 2) mutants revealed by quantitative characterization, suggest essentially equivalent functions of all four PpROPs in developing protonemata. However, cell and rhizoid length, as well as protonemata expansion, were reduced to a remarkably variable extent in *rop* ^3xKO^ mutants expressing distinct single PpROP family members (Fig. 2a,c–e), indicating that these proteins may differ in their ability to promote tip growth. Alternatively, the observed phenotypic variability of *rop* ^3xKO^ mutants may be caused by: differential up‐ or downregulation of the expression of the remaining Pp*ROP* gene after the disruption of the other members of the Pp*ROP* gene family; different genetic backgrounds resulting from multiple rounds of gene replacement based on homologous recombination; and/or by the expression of variable sets of selectable marker genes resulting from this procedure.

**Fig. 3:**
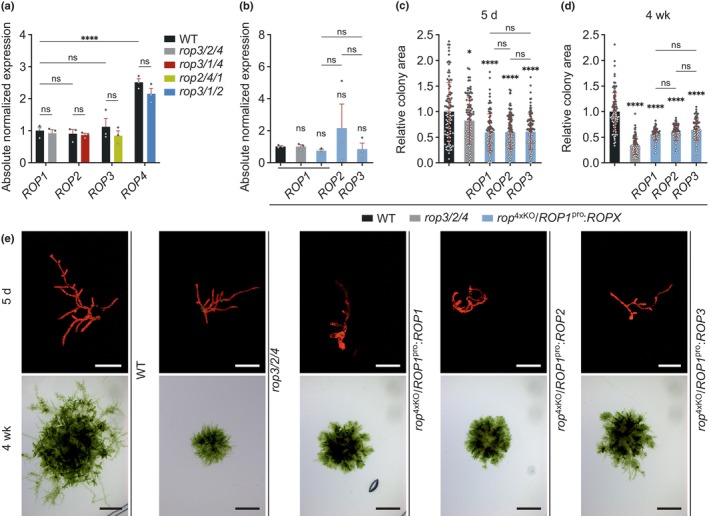
Pp*ROP*s are not altered in their gene expression in *rop* ^3xKO^ mutants and redundantly complement the *rop* ^4xKO^ mutant. (a, b) Reverse transcription quantitative polymerase chain reaction (RT‐qPCR) of expression levels of Pp*ROP*s in 1‐wk‐old *Physcomitrium patens* protonemata cultivated through homogenization (Supporting Information Table S2) with the indicated genotype compared with the wild‐type (WT): *rop* ^3xKO^ mutants (a); *rop* ^4xKO^ mutant complemented with the coding and 3′ untranslated region (UTR) sequence of Pp*ROP1* (Pp*ROP4* identical amino acid sequence) or Pp*ROP2* or Pp*ROP3* downstream of the Pp*ROP1* promoter (*rop* ^4xKO^/*ROP1* ^pro^:*ROPX*) (b). Absolute transcript levels were determined based on standard curves using the value obtained for one WT replicate of Pp*ROP1* as a calibrator (relative expression = 1). Bars, mean of three biological replicates; error bars, SE of the mean. The experiment was performed two times with consistent results. (c–e) Average size (area) of 5‐d‐old protonemata (c) or 4‐wk‐old colonies (d) regenerated from protoplasts with the indicated genotypes WT, *rop3/2/4*, *rop* ^4xKO^/*ROP1* ^pro^:*ROPX,* as determined based on microscopic imaging of Chl autofluorescence (e, 5 d) or pictures recorded using a stereo microscope (e, 4 wk). The mean value of WT was used as a calibrator (relative area = 1). *n* = 100 (c) or *n* = 80 (d) colonies per genotype were measured in three independent experiments. Error bars: SD, dots represent individual data points. Bars: 250 μm (e upper row, 5 d), 5 mm (e lower row, 4 wk). Statistical analysis by two‐way ANOVA/Bonferroni's test (a) or one‐way ANOVA/Tukey's test (b–d). Pairwise comparisons to WT and relevant pairwise comparisons are displayed; all others provided in Table S5: ^ns^, *P* > 0.05 (not significant); *, *P* ≤ 0.05; ****, *P* ≤ 0.0001.

RT‐qPCR analysis established that in all four *rop* ^3xKO^ mutants the remaining Pp*ROP* gene was expressed at the same level as the corresponding gene in the WT background (Fig. 3a). This demonstrates that the phenotypic variability displayed by *rop* ^3xKO^ mutants is not caused by differential feed‐back control of transcript levels within the Pp*ROP* gene family. To determine whether differences in genetic background or marker gene expression may have contributed to this variability, the activity of all PpROP family members was directly compared by phenotypically characterizing *rop* ^4xKO^/*ROP1* ^pro^:*ROPX* lines, which expressed either PpROP1, PpROP2 or PpROP3 under the control of the Pp*ROP1* promoter in an identical *rop* ^4xKO^ background (Fig. S1a–c). PpROP4 was not included into this analysis because it shares 100% amino acid identity with PpROP1. RT‐qPCR analysis confirmed that each of the *ROP1* ^pro^:*ROPX* genes was expressed in the *rop* ^4xKO^ background at a similar level as the endogenous Pp*ROP1* gene in the *rop3/2/4* mutant or in WT protonemata (Fig. 3b). Size and morphology of 5‐d‐old protonemata as well as of 4‐wk‐old colonies formed by all *rop* ^4xKO^/*ROP1* ^pro^:*ROPX* lines were found to be indistinguishable (Fig. 3c–e), establishing that all four PpROPs are equally capable of promoting tip growth.

### Disruption of all four Pp*ROP* genes completely abolishes polarized cell expansion and affects cell division

**Fig. 4:**
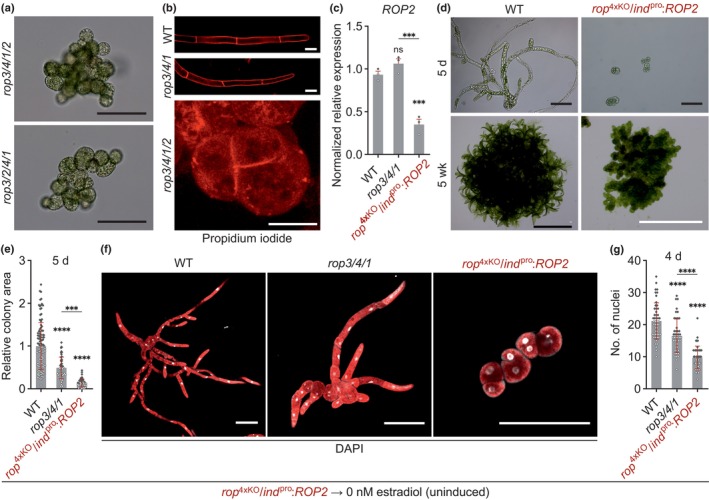
Loss of all Pp*ROP*s affects cell division and eliminates tip growth. Analysis of the *rop* ^4xKO^ phenotype was performed using *Physcomitrium patens* protonemata or 5‐wk‐old colonies cultivated on media listed in Supporting Information Table S2 with the indicated genotypes: wild‐type (WT); *rop3/4/1*; *rop3/4/1/2*, *rop3/2/4/1* (*rop* ^4xKO^); and *rop* ^4xKO^/*ind* ^pro^:*ROP2* uninduced (conditional complementation of *rop* ^4xKO^ with the coding and 3′ untranslated region (UTR) sequence of Pp*ROP2* downstream of the inducible β‐estradiol promoter). (a–c) One‐week‐old protonemata cultivated through homogenization. (a) Bright‐field micrographs of *rop* ^4xKO^ mutants. Bars, 100 μm. (b) Confocal imaging of the cell division plane using projections of serial confocal optical sections of protonemata stained with propidium iodide. Bars, 25 μm. (c) Reverse transcription quantitative polymerase chain reaction (RT‐qPCR) of relative Pp*ROP2* transcript levels determined according to the $2^{-\Delta \Delta C_{T}}$ method, using the value obtained for one WT replicate as a calibrator (relative expression = 1). Bars: mean of three biological replicates. The experiment was repeated two times with consistent results. (d–g) Analysis of the *rop* ^4xKO^ phenotype using 4‐ or 5‐d‐old protonemata or 5‐wk‐old colonies regenerated from protoplasts with the indicated genotypes. *rop* ^4xKO^/*ind* ^pro^:*ROP2* protoplasts were regenerated without β‐estradiol resembling the *rop* ^4xKO^ phenotype. (d) Bright‐field images from 5‐d‐old protonemata (upper panel) and images of 5‐wk‐old colonies (lower panel) were recorded using a stereo microscope. Bars: 100 μm (upper panel), 10 mm (lower panel, black bar), and 500 μm (lower panel, white bars). (e) Average size (area) of 5‐d‐old protonemata regenerated from protoplasts as determined based on microscopic imaging of Chl autofluorescence using the mean value of WT as calibrator (relative area = 1). *n* = 50 colonies per genotype measured in three independent experiments. (f, g) Cell division rate in 4‐d‐old protonemata was analyzed by recording Z‐stacks of confocal optical sections showing ′,6‐diamidino‐2‐phenylindole (DAPI) staining (white) and Chl autofluorescence (red) (maximum projections) Bars, 100 μm. (f) and determination of the average nuclei number (g). *n* = 50 colonies per genotype measured in three independent experiments. (c, e, g) Error bars: SE of the mean (c), SD (e, g), dots represent individual data points. Statistical analysis by one‐way ANOVA/Tukey's test (pairwise comparisons to WT and relevant pairwise comparisons are displayed; all others see Table S5): ^ns^, *P* > 0.05 (not significant); ***, *P* ≤ 0.001; ****, *P* ≤ 0.0001.

Two different *rop* ^4xKO^ lines independently generated based on homologous recombination (Figs S1a–c, S2a,b) displayed the same phenotype (Fig. 4a) as previously described *P. patens* mutants, in which the expression of all four Pp*ROP* genes was disrupted either by RNAi/microRNA‐mediated downregulation (Burkart *et al*., 2015; Bao *et al*., 2022), by CRISPR/Cas9‐mediated knockout (Cheng *et al*., 2020), or by a combination of these approaches (Yi & Goshima, 2020). Mutants entirely or nearly completely lacking PpROP activity form tiny colonies of irregularly shaped cells (Fig. 4a). In contrast to cells containing at least one functional PpROP gene (*rop* ^3xKO^), protonemal *rop* ^4xKO^ cells were unable to undergo normal polarization, maintain parallel orientation of cell division planes required for the development of linear filaments, or directionally expand (Fig. 4a,b). When cultured for prolonged periods of time, *rop* ^4xKO^ colonies continued to slowly grow based on cell division but failed to differentiate and did not develop gametophores. Interestingly, *rop* ^4xKO^ lines essentially phenocopy *P. patens* knockout mutants lacking geranylgeranyltransferase type I activity (Thole *et al*., 2014), suggesting that PpROPs require prenylation by this activity to be functional (Bao *et al*., 2022).

In addition to the defects described in the previous paragraph, quadruple Pp*ROP* RNAi knock‐down mutants were demonstrated to also display reduced cell adhesion and altered cell wall composition (Burkart *et al*., 2015; Bao *et al*., 2022). Consistent with these observations, protoplasts could not be isolated from colonies formed by the two different *rop* ^4xKO^ mutants described here, hampering the comparative quantitative phenotypic characterization of these mutants. To circumvent this issue, conditionally complemented *rop* ^4xKO^/*ind* ^pro^:*ROP2* lines were generated, which expressed PpROP2 under the control of an estradiol‐inducible promoter (Kubo *et al*., 2013) in a *rop* ^4xKO^ background (Fig. S2c,d). In the absence of estradiol, these lines produced Pp*ROP2* transcripts at substantially lower levels as compared to the *rop3/4/1* mutant (Fig. 4c) and displayed a typical *rop* ^4xKO^ phenotype after 5 d and after 5 wk in culture (Fig. 4d). Adding 1 nM estradiol to the culture medium resulted in partial complementation of the *rop* ^4xKO^ phenotype, which enabled protoplast isolation.

Quantitative comparison of protoplast‐derived 5‐d‐old protonemata growing on estradiol‐free culture medium confirmed that *rop* ^4xKO^/*ind* ^pro^:*ROP2* colonies displaying a *rop* ^4xKO^ phenotype were significantly smaller than *rop3/4/1* colonies (Fig. 4e), which showed the strongest growth defects of all *rop* ^3xKO^ mutants when compared to the WT (Fig. 2c,d,f,g). Remarkably, counting DAPI‐labeled nuclei in protoplast‐derived protonemata grown for 4 d in the absence of estradiol (Fig. 4f) established that the observed stepwise reduction of the size of WT, *rop3/4/1*, and *rop* ^4xKO^/*ind* ^pro^:*ROP2* (*rop* ^4xKO^ phenotype) protonemata (Fig. 4e) is a consequence not only of inhibited cell expansion but also of a similar stepwise decrease in the rate of cell division (Fig. 4g). The cell division rate of apical protonemal cells is enhanced by caulonema differentiation (Cove & Knight, 1993; Jang & Dolan, 2011) and possibly also, as observed in other cell types (Fantes & Nurse, 1977; Jones *et al*., 2017), by rapid cell expansion. The complete disruption of both these processes in *rop* ^4xKO^ mutants (Fig. 4) and the partial inhibition of cell expansion in *rop* ^3xKO^ mutants (Fig. 2), therefore, appear likely to be partially responsible for the reduced cell proliferation displayed by these mutants. However, like related mammalian RHO GTPases (Coleman *et al*., 2004), PpROPs may also directly target cell cycle regulation to enhance the rate of cell division, in addition to promoting cell expansion and regulating cell differentiation.

Comparing the phenotypes of *rop* ^4xKO^ mutants and of the four *rop* ^3xKO^ mutants expressing distinct individual PpROPs (Figs 2, 3, 4) establishes that each of these proteins alone (in the absence of the other three PpROPs) is capable not only of inducing polarization and directional expansion (albeit at a reduced rate) of protonemal cells, but also of supporting parallel cell division plane positioning required for filament formation, as well as of directly and/or indirectly promoting cell proliferation.

### Estradiol‐induced Pp*ROP2* expression at increasing levels progressively complements different defects displayed by *rop* ^4xKO^ mutants

**Fig. 5:**
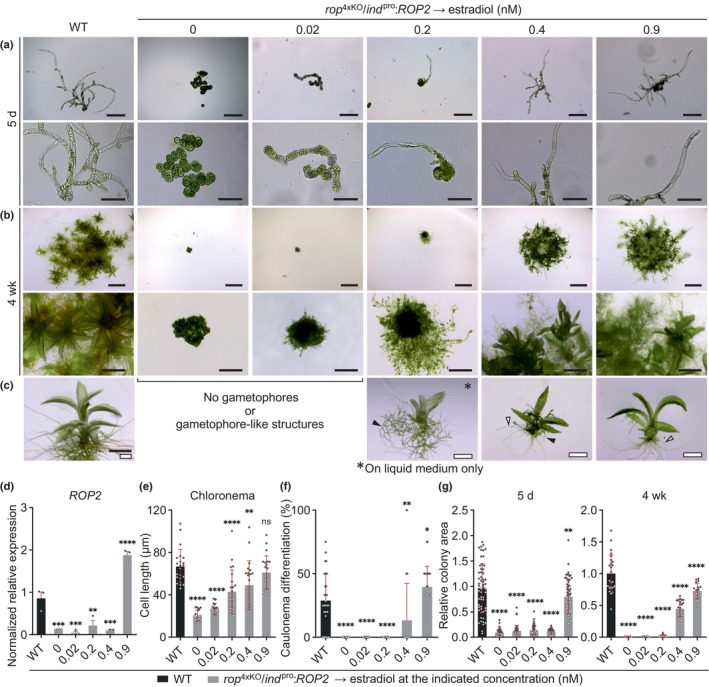
Titratable Pp*ROP2* expression rescues *rop* ^4xKO^. Five‐day‐old *Physcomitrium patens* protonemata or 4‐wk‐old colonies with the indicated genotypes were regenerated from protoplasts on agar‐solidified media (unless otherwise noted, see Supporting Information Table S2): wild‐type (WT) and *rop* ^4xKO^/*ind* ^pro^:*ROP2* (conditional complementation of *rop* ^4xKO^ with the coding and 3′ untranslated region (UTR) sequence of Pp*ROP2* downstream of the inducible β‐estradiol promoter) treated with β‐estradiol at the indicated concentrations. Five‐day‐old protonemata were imaged by transmitted‐light bright‐field microscopy (a). Images of 4‐wk‐old colonies (b) and of gametophore‐like structures or regular gametophores (c) were acquired using a stereo microscope. *rop* ^4xKO^/*ind* ^pro^:*ROP2* colonies did not develop gametophores after 4 wk in the absence or with minimal Pp*ROP2* expression (0 nM, 0.02 nM β‐estradiol). Treatment with 0.2 nM β‐estradiol generated gametophore‐like structures only in liquid BCD medium (asterisk). At ≥ 0.4 nM β‐estradiol regular gametophores with reduced size developed on BCD medium solidified with agar. Closed triangles indicate protonemata, open triangles indicate rhizoids. Bars, 200 μm (1^st^ row), 100 μm (2^nd^ row), 5 mm (3^rd^ row), 1 mm (4^th^ row), 1 mm (5^th^ row, black bar), and 500 μm (5^th^ row, white bars). (d) Relative Pp*ROP2* transcript levels were determined in 7‐d‐old (WT, *rop* ^4xKO^/*ind* ^pro^:*ROP2* treated with 0.9 nM β‐estradiol) or 10‐d‐old protonemata (*rop* ^4xKO^/*ind* ^pro^:*ROP2* treated with ≤ 0.4 nM β‐estradiol) according to the $2^{-\Delta \Delta C_{T}}$ method, using the value obtained for one WT replicate as calibrator (relative expression = 1). Bars: mean of three biological replicates. The experiment was repeated two times with consistent results. Average length of subapical chloronemal cells (e) and average percentage of caulonema differentiation (f) as determined by microscopic observation of 5‐d‐old protonemata. *n* = 22 cells per genotype (e), or *n* = 30 colonies per genotype (f) measured in three independent experiments. (g) Average size (area) of 5‐d‐old protonemata or 4‐wk‐old colonies using the mean value of WT as calibrator (relative area = 1). *n* = 60 colonies per genotype (5 d), or *n* = 22 colonies per genotype (4 wk) measured in 3 independent experiments. Error bars: SE of the mean (d), SD (e–g). Dots represent individual data points. Statistical analysis by one‐way ANOVA/Tukey's test (pairwise comparisons to WT are displayed, all others see Table S5): ^ns^, *P* > 0.05 (not significant); *, *P* ≤ 0.05; **, *P* ≤ 0.01; ***, *P* ≤ 0.001; ****, *P* ≤ 0.0001.

Data presented in Figs 1, 2, 3, 4 strongly suggest that the four nearly identical PpROPs are functionally redundant, and increasing levels of total PpROP activity are required for different important processes during protonemal development. To further support these findings, the phenotypes of *rop* ^4xKO^/*ind* ^pro^:*ROP2* protonemata were investigated, in which Pp*ROP2* expression was gradually induced by increasing estradiol concentrations in the culture medium. Estradiol concentrations eliciting striking phenotypic alterations were selected for this analysis (Fig. 5a–c). Control experiments confirmed that estradiol at these concentrations did not affect the development of WT protonemata (Fig. S8).

At concentrations of up to 0.4 nM, estradiol‐induced Pp*ROP2* expression remained much lower as compared to the WT expression level of this gene (Fig. 5d), which also corresponds to the level of total Pp*ROP* gene expression in *rop3/4/1* and other *rop* ^3xKO^ mutants (Figs 3a, S4). A linear correlation between estradiol concentrations of up to 0.4 nM and resulting minimal levels of Pp*ROP2* expression, which were near the detection limit, could not be established based on RT‐qPCR analysis (Fig. 5d). Pp*ROP2* expression induced by 0.9 nM estradiol was *c*. 2.3× stronger than in WT protonemata (Fig. 5d) and thus reached a level comparable to total Pp*ROP* gene expression in *rop* ^2xKO^ mutants (2–3× WT Pp*ROP2* expression level; Fig. S4). Unfortunately, higher estradiol concentrations failed to further enhance Pp*ROP2* expression to levels comparable to total Pp*ROP* gene expression in normally developing *rop* ^1xKO^ or WT protonemata (3–4× or 5× WT Pp*ROP2* expression level, respectively; Fig. S4).

As already shown above (Fig. 4d), in the absence of estradiol, *rop* ^4xKO^/*ind* ^pro^:*ROP2* protonemata displayed a typical *rop* ^4xKO^ phenotype after 5 d and after several weeks in culture (Fig. 5a–c, second column). Remarkably, induction of Pp*ROP2* expression at the lowest level by 0.02 nM estradiol partially restored cell polarization and parallel cell division plane positioning required for filament formation (Fig. 5a,b), without substantially promoting cell elongation or colony expansion (Fig. 5a,b,e,g; Table S6) or supporting caulonema differentiation (Fig. 5a,f). These observations demonstrate that cell polarization and parallel cell division plane positioning can be uncoupled from tip growth and that these processes depend on different levels of total PpROP activity.

On solid culture medium containing 0.2 nM estradiol, at low frequency, chloronemal filaments evidently elongating based on tip growth were formed (Fig. 5a,b; Table S6). However, this process was not fully restored, as subapical cells in these filaments were much shorter than corresponding WT cells (Fig. 5e), and colony growth remained strongly restricted (Fig. 5a,b,g). Caulonema differentiation (Fig. 5a,f) or gametophore formation (Fig. 5b) was not observed under these conditions. Interestingly, colonies growing for 4 wk in liquid medium containing estradiol at the same concentration, and displaying an otherwise indistinguishable phenotype, frequently developed small gametophore‐like structures, which contained two to four rudimentary phyllids but failed to form stems or rhizoids (Fig. 5c). This finding is consistent with previous reports demonstrating that liquid culture can promote gametophore formation (Reski & Abel, 1985). As similar gametophore‐like structures were never formed by *rop* ^4xKO^/*ind* ^pro^:*ROP2* colonies growing in liquid medium containing estradiol at lower concentrations, PpROP activity at minimal levels appears to be able to induce the formation of gametophore‐like structures even in the absence of caulonema differentiation. Furthermore, PpROP activity at these levels evidently is sufficient for the development of rudimentary phyllids.

Pp*ROP2* expression induced by 0.4 nM estradiol further promoted directional cell expansion (Fig. 5a,b,e; Table S6) and partially rescued caulonema differentiation (Fig. 5a,f). This led to a substantial increase in the size of 4‐wk‐old colonies (Fig. 5b,g) and was accompanied by the frequent development of regular gametophores with a reduced size and severely stunted rhizoids on solid culture medium (Fig. 5c). However, the length of subapical protonemal cells (Fig. 5a,e), the rate of caulonema differentiation (Fig. 5f), as well as the size of 5‐d‐old and 4‐wk‐old colonies (Fig. 5g), remained significantly decreased. Interestingly, essentially the same developmental defects are shown by *rop* ^3xKO^ mutants (Fig. 2), although the phenotype of these mutants appears to be somewhat weaker, as they display caulonema differentiation at WT rates, which are significantly reduced only in comparison with *rop* ^2xKO^ mutants.

Finally, *rop* ^4xKO^/*ind* ^pro^:*ROP2* protonemata growing in medium containing 0.9 nM estradiol essentially phenocopied *rop* ^2xKO^ mutants (Figs 1, 2, 3, 4, 5; Table S6), which, as discussed above, exhibit similar levels of total Pp*ROP* gene expression. As compared to WT protonemata, *rop* ^4xKO^/*ind* ^pro^:*ROP2* protonemata expressing Pp*ROP2* at this level of induction displayed significantly enhanced caulonema differentiation (Fig. 5f), while subapical cell length in 5‐d‐old protonemata was not detectably affected (Fig. 5e), and colony size after 5 d or after 4 wk in culture, as well as gametophore growth, were only weakly reduced (Fig. 5a–c,g).

Data presented in this section demonstrate that PpROP2, a single member of the PpROP family, can effectively complement different severe defects displayed by *rop* ^4xKO^ mutants. Partially interdependent developmental processes disrupted in these mutants were progressively restored by increasing levels of estradiol‐induced Pp*ROP2* expression, indicating that these processes require distinct levels of total PpROP activity, rather than specific individual ROPs. Listed in the order of their increasing requirement for total PpROP activity, the following processes were restored by estradiol‐induced PpROP2 expression: (1) cell polarization and parallel cell division plane positioning required for filament formation; (2) tip growth; (3) development of gametophore‐like structures or small gametophores; and (4) caulonema differentiation, which is tightly correlated with enhanced cell expansion (Jang & Dolan, 2011) and appears to depend on this process, as discussed above.

### Pp*ROP* overexpression inhibits caulonema differentiation and depolarizes directional cell expansion

**Fig. 6:**
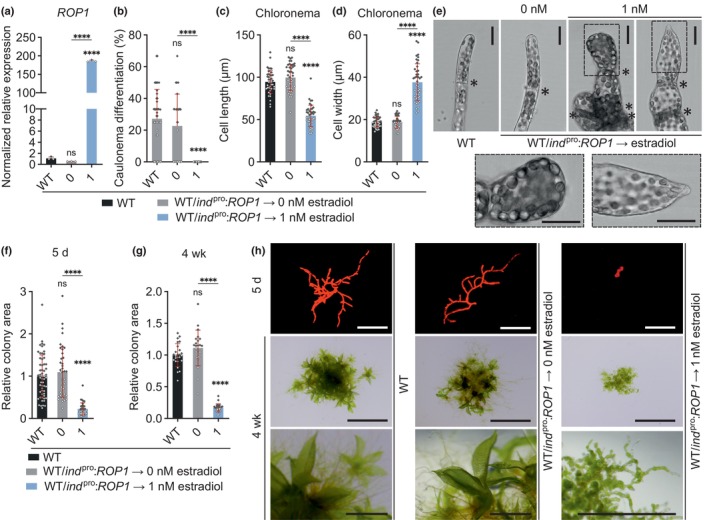
Pp*ROP* overexpression deregulates tip growth and caulonema differentiation. One‐week‐old *Physcomitrium patens* protonemata cultivated through homogenization (a) or 5‐d‐old protonemata or 4‐wk‐old colonies regenerated from protoplasts (b–h) using media listed in Supporting Information Table S2 with the indicated genotypes: wild‐type (WT); WT/i*nd* ^*pro*^:*ROP1* (overexpression of the Pp*ROP1* coding sequence and 3′ untranslated region (UTR)) in WT using the inducible β‐estradiol promoter. (a) Relative transcript levels of Pp*ROP1* were determined according to the $2^{-\Delta \Delta C_{T}}$ method using the value obtained for one WT replicate as calibrator (relative expression level = 1). Bars: mean of three biological replicates. The experiment was repeated two times with consistent results. (b–d) Average percentage of caulonema differentiation as determined by microscopic observation (b) or average subapical chloronemal cell length (c) or cell width (d) in filaments with at least three cells of 5‐d‐old protonemata. *n* = 42 cells per genotype measured in three independent experiments. (e) Bright‐field micrographs of filament tips of 5‐d‐old protonemata with the indicated genotypes. Asterisks indicate the cell wall between neighboring cells, dotted rectangles indicate the magnified apical region. Bars, 25 μm. (f–h) Average size (area) of 5‐d‐old protonemata (f) or 4‐wk‐old colonies (g) using the mean value of WT as calibrator (relative area = 1) were determined based on microscopic imaging of Chl autofluorescence (h, first top row; 5 d) or pictures recorded using a stereo microscope (h, second and third rows; 4 wk). Bars, 500 μm (1^st^ top row), 5 mm (second row), 1 mm (third row). *n* = 35 (f) or *n* = 23 (g) colonies per genotype. The experiment was repeated two times, and the results from one representative experiment are shown here. (a–d, f, g) Error bars: SE of the mean (a), SD (b–d, f, g). Dots represent individual data points. Statistical analysis by one‐way ANOVA/Tukey's test (pairwise comparisons are displayed): ^ns^, *P* > 0.05 (not significant); ****, *P* ≤ 0.0001.

To investigate the effects of PpROP overexpression, a WT/*ind* ^pro^:*ROP1* line was established (Fig. S1d,e), which exhibits massive estradiol‐inducible Pp*ROP1* overexpression in the WT background. In the absence of estradiol, protoplast‐derived protonemata of this line expressed Pp*ROP1* at WT levels (Fig. 6a) and displayed no developmental defects (Fig. 6b–h). Growing these protonemata on solid medium containing 1 nM estradiol resulted in Rp*ROP1* expression at a > 150‐fold higher level (Fig. 6a), which roughly corresponds to a 30‐fold increase in total Pp*ROP* gene expression as compared to WT protonemata (total Pp*ROP* gene expression *c*. 5‐fold stronger than Pp*ROP1* expression; Fig. S4). Estradiol at this concentration did not affect Pp*ROP1* expression or developmental processes in WT protonemata (Fig. S8).

As discussed above, PpROP activity was concluded to suppress caulonema differentiation based on the observation that this process is enhanced in otherwise essentially normally developing *rop* ^2xKO^ protonemata (Figs 1, S6a). Further supporting this conclusion, Pp*ROP1* overexpression effectively disrupted caulonema differentiation (Fig. 6b). Moreover, quantitative analysis of protoplast‐derived WT/*ind* ^pro^:*ROP1* protonemata revealed that high‐level Pp*ROP1* overexpression strongly reduced the length and enhanced the width of subapical cells in chloronemal filaments (Fig. 6c–e), which indicates massive depolarization of tip growth displayed by adjacent apical cells. Interestingly, PpPOP1 overexpression caused the apical dome of these cells to oscillate at irregular intervals between a wide‐flattened and a narrow‐pointed morphology (Fig. 6e). Together, inhibited caulonema differentiation and defective cell expansion strongly reduced the size of Pp*ROP1*‐overexpressing 5‐d‐old protonemata and of 4‐wk‐old colonies (Fig. 6f–h). Remarkably, WT/*ind* ^pro^:*ROP1* protonemata never developed gametophores when growing on solid or in liquid medium containing 1 nM estradiol, demonstrating that PpPOP1 overexpression effectively blocked the formation of these structures (Fig. 6h).

In summary, consistent with and further defining PpROP functions identified by the phenotypic characterization of *rop* ^KO^ mutants, PpROP1 overexpression prevented caulonema differentiation, effectively depolarized cell expansion at the tip of protonemal filaments, strongly reduced protonemal growth, and completely blocked gametophore formation. As discussed above, caulonema differentiation is tightly correlated with enhanced cell expansion, indicating interdependence between these processes (Jang & Dolan, 2011). PpROP1 overexpression may, therefore, inhibit caulonema differentiation, at least in part, by restricting cell expansion. Similarly, chloronemal filaments were proposed to lack competence for gametophore formation (Cove & Knight, 1993; Schumaker & Dietrich, 1997; Brun *et al*., 2003; Harrison *et al*., 2009), indicating that PpROP overexpression may indirectly block gametophore formation by inhibiting caulonema differentiation. Consistent with this interpretation, increasing ROP activity progressively stimulates the formation of gametophore‐like structures and the development of gametophores (Figs 2, 5), suggesting that ROP activity promotes rather than inhibits these processes.

### PpROP functions in fundamental developmental processes depend on GDP/GTP cycling

**Fig. 7:**
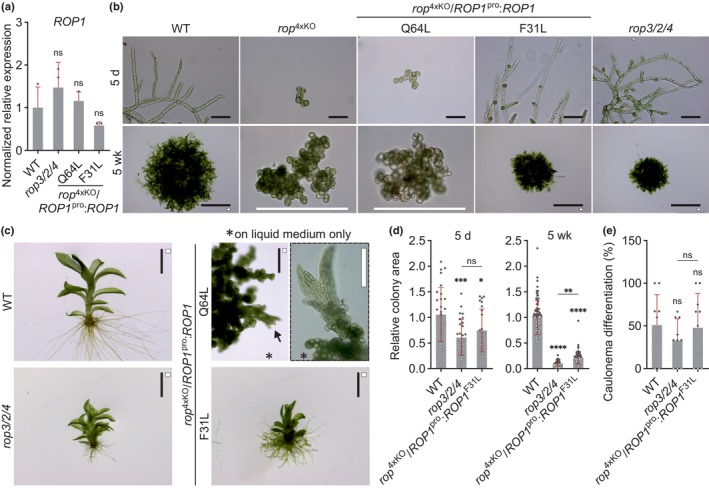
GDP/GTP cycling is essential for most PpROP functions but not gametophore initiation. Graphs and images based on *Physcomitrium patens* protonemata or gametophores or colonies using culture conditions (Table S2) with the indicated genotypes: wild‐type (WT); *rop2/3/4*; *rop* ^4xKO^; *rop* ^4xKO^/*ROP1* ^pro^:*ROP1* ^Q64L^ (complementation of *rop* ^4xKO^ with the Pp*ROP1* coding and 3′ untranslated region (UTR) sequence containing a constitutive active mutation (Q64L) downstream of the endogenous Pp*ROP1* promoter); and *rop* ^4xKO^/*ROP1* ^pro^:*ROP1* ^F31L^ (complementation of *rop* ^4xKO^ with the Pp*ROP1* coding and the 3′ UTR sequence containing a fast‐cycling mutation (F31L) downstream of the endogenous Pp*ROP1* promoter). (a) Relative transcript levels of Pp*ROP1* in 1‐wk‐old protonemata cultivated through homogenization according to the $2^{-\Delta \Delta C_{T}}$ method, using the value obtained for one WT replicate as a calibrator (relative expression = 1). Bars: mean of three biological replicates. The experiment was repeated two times with consistent results. (b–e) Five‐day‐old protonemata or 5‐wk‐old colonies regenerated from protoplasts (WT, *rop2/3/4*, *rop* ^4xKO^/*ROP1* ^pro^:*ROP1* ^F31L^) or cultivated through homogenization (*rop* ^4xKO^, *rop* ^4xKO^/*ROP1* ^pro^:*ROP1* ^Q64L^). (b) Images of 5‐d‐old protonemata recorded using bright‐field microscopy (upper row) and images of 5‐wk‐old colonies obtained using a stereo microscope (WT, *rop2/3/4*, *rop* ^4xKO^/*ROP1* ^pro^:*ROP1* ^F31L^) or bright‐field microscopy (*rop* ^4xKO^, *rop* ^4xKO^/*ROP1* ^pro^:*ROP1* ^Q64L^) (lower row). Bars, 100 μm (upper row), 10 mm (black bars, lower row), and 400 μm (white bars, lower row). (c) Images of 5‐wk‐old gametophores cultivated on solid BCD medium except *rop* ^4xKO^/*ROP1* ^pro^:*ROP1* ^Q64L^ that developed gametophore‐like structures only in liquid BCD medium (asterisk) were recorded using a stereo microscope. Arrow indicates a gametophore‐like structure used for magnification using bright‐field microscopy (dotted rectangle). Bars, 2 mm (black bars) and 200 μm (white bars). (d) Average size (area) of 5‐d‐old protonemata or 5‐wk‐old colonies normalized to the mean value of WT (relative area = 1). *n* = 30 colonies per genotype; the experiment was repeated three times with consistent results (5 d) or *n* = 81 colonies per genotype measured in three independent experiments (5 wk). (e) Average percentage of caulonema differentiation in 5‐d‐old protonemal filaments with at least three cells was determined by microscopic observation. *n* = 10 colonies per genotype. The experiment was repeated three times with consistent results. (a, d, e) Error bars: SE of the mean (a), SD (d, e), dots represent individual data points. Statistical analysis by one‐way ANOVA/Tukey's test (pairwise comparisons to WT and relevant pairwise comparisons are displayed, all others see Table S5). ^ns^, *P* > 0.05 (not significant); *, *P* ≤ 0.05; **, *P* ≤ 0.01; ***, *P* ≤ 0.001; ****, *P* ≤ 0.0001. GTP, guanosine triphosphate.

Highly conserved amino acid residues can be mutated to alter the intrinsic rate of GDP/GTP cycling displayed by RHO GTPases (Aspenström, 2019). These proteins are locked in the GTP‐bound state by mutations corresponding to the Q61L exchange in H‐RAS, which disrupt GTPase activity (GTP‐locked). By contrast, mutations equivalent to the F28L exchange in H‐RAS reduce nucleotide‐binding affinity and enhance intrinsic GDP/GTP exchange (fast‐cycling). Fast‐cycling RHO mutants are preferentially GTP‐bound at high GTP/GDP concentration ratios typically observed in the cytoplasm of eukaryotic cells. Like GTP‐locked mutants, fast‐cycling mutants are therefore considered to be constitutively active. To assess the importance of GDP/GTP cycling for PpROP functions in protonemal development, *rop* ^4xKO^/*ROP1* ^pro^:*ROP1* ^Q64L^ and *rop* ^4xKO^/*ROP1* ^pro^:*ROP1* ^F31L^ lines were generated, which expressed either GTP‐locked PpROP1^Q64L^ or fast‐cycling PpROP1^F31L^ under the control of the Pp*ROP1* promoter in the same *rop* ^4xKO^ background (Fig. S1a–c). Based on RT‐qPCR analysis of 1‐wk‐old protonemata, lines expressing mutated Pp*ROP1* genes at the same level as Pp*ROP1* expression in WT plants and in *rop3/2/4* mutants were identified (Fig. 7a) and phenotypically characterized (Fig. 7b–e).

Even after prolonged culture on solid medium, *rop* ^4xKO^/*ROP1* ^pro^:*ROP1* ^Q64L^ colonies displayed a characteristic *rop* ^*4x*KO^ phenotype (Fig. 7b). As this phenotype includes cell wall defects preventing protoplast isolation, the *rop* ^4xKO^/*ROP1* ^pro^:*ROP1* ^Q64L^ colonies shown were grown from tissue explants. This remarkable observation established that GDP/GTP cycling is strictly required for PpROP functions in fundamental developmental processes, including cell polarization and parallel division plane positioning required for filament formation. It remains to be determined whether GDP/GTP exchange is also essential for ROP functions in directional cell expansion and other processes, which appear to directly or indirectly depend on cell polarization and/or parallel division plane positioning. Interestingly, like *rop* ^4xKO^/*ind* ^pro^:*ROP2* colonies in the presence of estradiol at low concentration (0.2 nM; Fig. 5c), *rop* ^4xKO^/*ROP1* ^pro^:*ROP1* ^Q64L^ colonies frequently formed gametophore‐like structures exclusively when grown in liquid medium (Fig. 7c). The induction of gametophore formation in the absence of caulonema differentiation, as well as the promotion of rudimentary phyllid development by low‐level PpROP activity, therefore does not require GDP/GTP cycling.

Protoplast‐derived 5‐d‐old and 5‐wk‐old *rop* ^4xKO^/*ROP1* ^pro^:*ROP1* ^F31L^ and *rop3/2/4* protonemata displayed very similar phenotypes (Fig. 7b). As compared to WT protonemata, colony size was similarly reduced (Fig. 7d), caulonema differentiation was not significantly affected (Fig. 7e), and gametophores with small but morphologically essentially normal leafy shoots and severely stunted rhizoids were formed (Fig. 7c). These data establish that, in contrast to GTP‐locked PpROP1^Q64L^, which is severely functionally impaired, fast‐cycling PpROP1^F31L^ displays essentially the same ability as WT PpROP1 to promote protonemal development. However, it remains to be demonstrated that PpROP1^F31L^ is also capable of inhibiting caulonema differentiation when expressed at endogenous levels in a *rop* ^2xKO^ mutant, or upon overexpression.

### Overexpression of heterologous RHO GTPases in the *rop* ^4xKO^ background suggests limited evolutionary conservation of PpROP downstream signaling

**Fig. 8:**
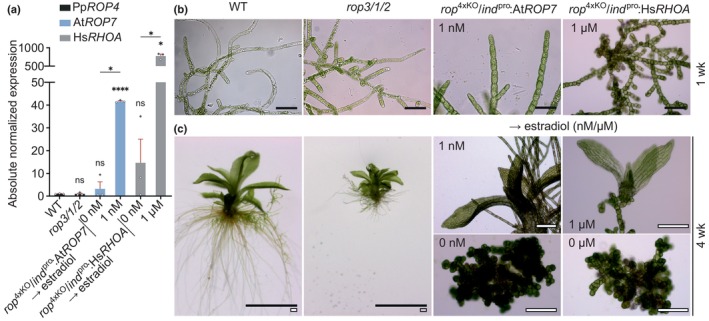
At*ROP7* or Hs*RHOA* overexpression partially rescues Pp*ROP*s. One‐week‐old *Physcomitrium patens* protonemata or 4‐wk‐old colonies were cultivated through homogenization using media described in Supporting Information Table S2 with the indicated genotypes: wild‐type (WT); *rop1/2/3*; and *rop* ^4xKO^/*ind* ^pro^:At*ROP7* or *rop* ^4xKO^/*ind* ^pro^:Hs*RHOA* (complementation of *rop* ^4xKO^ with the coding sequence of At*ROP7* or Hs*RHOA* downstream of the β‐estradiol‐inducible promoter). (a) Absolute transcript levels of Pp*ROP4*, At*ROP7*, or Hs*RHOA* were determined in 1‐wk‐old protonemata with the indicated genotype based on standard curves, using the value obtained for one WT replicate of Pp*ROP4* as calibrator (relative expression = 1). Bars: mean of three biological replicates. Error bars: SE of the mean. The experiment was repeated two times with consistent results. Statistical analysis by one‐way ANOVA/Tukey's test (pairwise comparisons to WT and relevant pairwise comparisons are displayed, all others see Table S5): ^ns^, *P* > 0.05 (not significant); *, *P* ≤ 0.05; ****, *P* ≤ 0.0001. (b, c) Bright‐field micrographs of 1‐wk‐old protonemata (b), or images of 4‐wk‐old gametophores recorded using a stereo microscope (WT, *rop1/2/3*) or protonemata or gametophore‐like structures using a bright‐field microscope (*rop* ^4xKO^/*ind* ^pro^:At*ROP7*, *rop* ^4xKO^/*ind* ^pro^:Hs*RHOA*) (c). Bars: 50 μm (b); 2 mm (black bars), and 200 μm (white bars) (c).

To test the ability of heterologous RHO GTPases to complement the total loss of PpROP activity, AtROP7, the closest Arabidopsis PpROP homolog (Eklund *et al*., 2010a), and human HsRHOA, one of the most extensively characterized RHO GTPases (Bishop & Hall, 2000), were overexpressed in the same *rop* ^4xKO^ background. To this end, *rop* ^4xKO^/*ind* ^pro^:At*ROP7* and *rop* ^4xKO^/*ind* ^pro^:Hs*RHOA* lines were generated, which expressed AtROP7 or HsRHOA under the control of an estradiol‐inducible promoter (Kubo *et al*., 2013). Most lines obtained displayed a typical *rop* ^4xKO^ phenotype when grown in the presence of estradiol at different concentrations. Based on RT‐qPCR analysis of 1‐wk‐old protonemata (Fig. 8a), lines exhibiting maximal At*ROP7* or Hs*RHOA* transcript levels upon estradiol induction were therefore identified and selected for phenotypic characterization (Fig. 8b,c). Maximal At*ROP7* and Hs*RHOA* transcript levels obtained were *c*. 40× or > 500× higher, respectively, than Pp*ROP4* transcript levels in WT plants and in *rop3/1/2* mutants (Fig. 8a), which roughly corresponds to 16× or > 200× higher transcript levels as compared to total Pp*ROP* gene expression in WT protonemata (*c*. 2.5× stronger than Pp*ROP4* expression; Fig. S4). Control experiments showed that estradiol at concentrations of 1 nM or 1 μM, which maximally induced At*ROP7* or Hs*RHOA* expression, respectively, did not affect developmental processes in WT protonemata (Fig. S8).

Low‐level At*ROP7* or Hs*RHOA* expression in the absence of estradiol did not detectably rescue the *rop* ^4xKO^ phenotype (Fig. 8a,c). By contrast, massive overexpression of either protein in the presence of estradiol complemented this phenotype to a similar extent (Fig. 8a–c) as estradiol‐induced Pp*ROP2* expression at minimal levels (0.02–0.2 nM; Fig. 5). Cell polarization and parallel cell division plane positioning required for filament formation were restored, whereas tip growth was not substantially promoted, and caulonema differentiation was never induced (Fig. 8b). Furthermore, even on solid culture medium, small gametophore‐like structures occasionally developed, which contained a few rudimentary phyllids, but unlike regular WT or *rop* ^3xKO^ gametophores, failed to form stems or rhizoids (Fig. 8c). These observations establish that massive overexpression of heterologous flowering plant or mammalian RHO GTPases can restore fundamental developmental processes in *rop* ^4xKO^ mutants, which require minimal levels of total PpROP activity. Consequently, PpROP downstream signaling controlling these processes appears to be evolutionarily conserved at least to some extent.

## Discussion

**Fig. 9:**
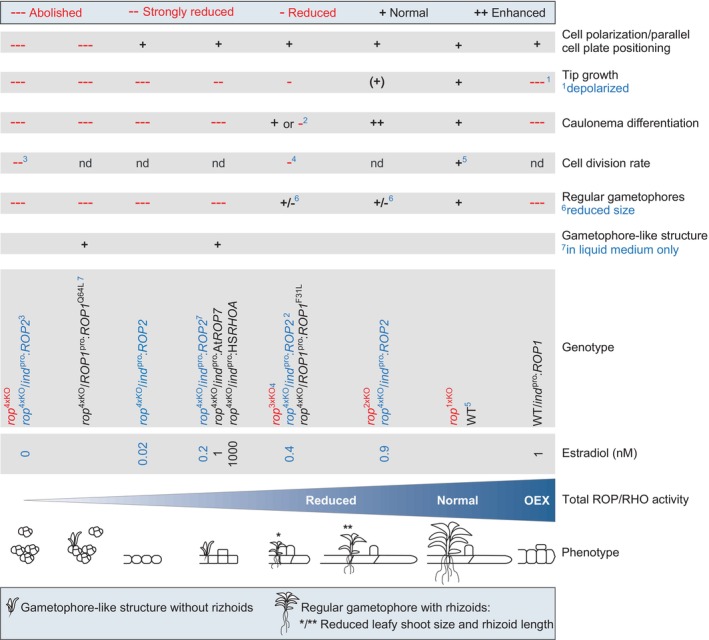
Different cellular and developmental process require distinct levels of total PpROP activity. Schematic overview of results obtained by comparative quantitative phenotypic characterization of *Physcomitrium patens* plants with the indicated genotypes: (a) wild‐type (WT; black font), (b) knockout mutants (red font) lacking single Pp*ROP* genes (*rop* ^1xKO^) or multiple such genes in all possible combinations (*rop* ^2xKO^, *rop* ^3xKO^, and *rop* ^4xKO^), (c) complemented *rop* ^4xKO^ mutants expressing Pp*ROP2* (blue font), Pp*ROP1* mutants (PpROP1^F31L^ and PpROP1^Q64L^; black font) or heterologous RHO GTPases (AtROP7 and HsRHOA; black font) either at endogenous (*ROP1* ^pro^) or at different β‐estradiol‐titrated levels (*ind* ^pro^; β‐estradiol concentrations indicated), and (d) lines displaying β‐estradiol‐induced PpROP1 overexpression (OEX) in the WT background (black font, *ind* ^pro^; β‐estradiol concentration 1 nM). PpROP1^Q64L^ expressed at endogenous level failed to complement *rop* ^4xKO^ mutants, but induced the formation of gametophore‐like structures. Minimal inducible PpROP2 expression (0.02 nM β‐estradiol) in *rop* ^4xKO^ mutants exclusively restored cell polarization and parallel cell plate positioning, resulting in the formation of filaments not exhibiting tip growth. Somewhat higher levels of ROP/RHO activity in *rop* ^4xKO^ mutants provided by low‐level inducible PpROP2 expression (0.2 nM β‐estradiol) or by massive overexpression of heterologous RHO GTPases (AtROP7, HsRHOA) induced the formation of gametophore‐like structures, and only in the case of PpROP2 expression weakly supported tip growth. PpROP activity in *rop* ^3xKO^ mutants, or in *rop* ^4xKO^ mutants complemented either by inducible PpROP2 expression (0.4 nM β‐estradiol) or by PpROP1^F31L^ expression at the endogenous level, further stimulated tip growth, enabled caulonema differentiation, and induced the formation of regular gametophores with small shoots and short rhizoids. Notably, *rop* ^3xKO^ and *rop* ^4xKO^/*ROP1* ^pro^:*ROP1* ^F31L^ protonemata displayed normal caulonema differentiation, which was reduced only in comparison with *rop* ^2xKO^ mutants. By contrast, caulonema differentiation was reduced also as compared to WT plants in *rop* ^4xKO^/*ROP1* ^pro^:*ROP2* protonemata growing on 0.4 nM estradiol. Further enhanced PpROP activity in *rop* ^2xKO^ mutants, and in *rop* ^4xKO^ mutants complemented by inducible PpROP2 expression (0.9 nM β‐estradiol), also induced in the formation of small regular gametophores, supported only marginally reduced tip growth and resulted in enhanced caulonema differentiation as compared to WT plants. While PpROP activity in *rop* ^1xKO^ mutants was sufficient for normal protonemal and gametophore development, high level‐inducible PpROP1 OEX in the WT background strongly inhibited tip growth, caulonema differentiation and gametophore formation. Furthermore, progressively reduced levels of PpROP activity in *rop* ^3xKO^ mutants and in uninduced *rop* ^4xKO^/Ind^pro^:*ROP2* lines (0 nM β‐estradiol), which display a *rop* ^4xKO^ phenotype, resulted in a gradually decreased rate of cell division as compared to WT plants (cells division rates of plants with other genotypes were not determined: nd). RHO, RAS homologous; ROP, RHO of plants.

PpROP functions in protonemal development were systematically investigated based on quantitative phenotypic characterization of: knockout mutants lacking single Pp*ROP* genes or multiple such genes in all possible combinations; complemented *rop* ^4xKO^ lines expressing WT PpROPs, constitutively active PpROP1 mutants, or heterologous RHO GTPases either at endogenous or different estradiol‐titrated levels; and lines displaying PpROP1 overexpression in the WT background. The results of these analyses firmly establish that the four nearly identical PpROPs, which are co‐expressed at similar levels in protonemata, are functionally redundant, and that different developmental processes depend on distinct levels of total PpROP activity, rather than on specific individual ROPs (Fig. 9).

### PpROP activity induces cell polarization and parallel division plain positioning at minimal levels, and increasingly promotes tip growth when further enhanced

Low levels of total PpROP activity induce cell polarization and parallel cell division plane positioning, which enables filament formation (Fig. 9). Consistent with an important role of PpROP activity in cell division plane positioning, defects in this process were also observed: in asymmetrically dividing cells in Arabidopsis roots overexpressing constitutively active mutant AtROP9 (Roszak *et al*., 2021) or in maize leaves lacking functional Zm*ROP2* and Zm*ROP9* genes (Humphries *et al*., 2011); during branch formation in *P. patens rop* ^3xKO^ protonemata (Yi & Goshima, 2020); and in different tissues of the liverwort *Marchantia polymorpha* after the disruption of the single Mp*ROP* gene expressed in this organism (Mulvey & Dolan, 2023a).

Filament formation restored by minimal levels of PpROP activity is not accompanied by substantial directional cell expansion. Tip growth of apical protonemal cells is induced at somewhat higher total PpROP activity and is further promoted by increasing levels of this activity until it reaches WT rates in *rop* ^1xKO^ mutants (Fig. 9). High‐level PpROP1 overexpression in *P. patens* protonemata massively depolarizes tip growth (Fig. 6c–e), consistent with similar but much weaker effects previously observed upon overexpression of a fluorescent PpROP2 fusion protein (Ito *et al*., 2014). In angiosperm pollen tubes and root hairs, excess ROP activity also strongly depolarizes tip growth and results in massive apical ballooning (Chen *et al*., 2003; Y. Gu *et al*., 2003; Klahre & Kost, 2006; Kost, 2008). However, unlike these other cell types, apical protonemal cells overexpressing PpROP activity do not simply swell at the tip but display complex growth behavior that warrants further investigation (Fig. 6e). Despite these somewhat different overexpression effects, ROP functions in the control of tip growth appear to be essentially conserved from mosses to flowering plants. Yeast and mammalian RHO GTPases also have well‐characterized functions in the regulation of directional cell expansion and related processes (Hall & Lalli, 2010; Ou & Yi, 2022).

### PpROP activity at WT and somewhat lower levels suppresses caulonema differentiation apparently via stimulation of the ROP effector PpRIC

As caulonema differentiation is enhanced (Fig. 1c) in normally growing 5‐d‐old *rop* ^2xKO^ protonemata (Figs 1a,b,d,f, S6a), this process appears to be suppressed by total PpROP activity at levels exhibited by *rop* ^1xKO^ and in WT protonemata (Fig. 9). This conclusion is further supported by recent findings demonstrating that caulonema differentiation is also promoted following PpROP inactivation by upstream regulators belonging to the PpGAP (GTPase activating proteins) or PpREN (ROP Enhancer) families (Ruan *et al*., 2023).

Like 5‐d‐old *rop* ^2xKO^ protonemata, *ric‐1* ^KO^ protonemata at the same developmental stage, which lack expression of the PpROP effector PpRIC, also displayed enhanced caulonema differentiation without showing substantial growth defects (Ntefidou *et al*., 2023). These observations strongly suggest that PpROP activity inhibits caulonema differentiation via PpRIC stimulation. PpRIC has been shown to suppress this process downstream of auxin‐induced changes in gene expression (Ntefidou *et al*., 2023). As previously demonstrated for *ric‐1* ^KO^ mutants (Ntefidou *et al*., 2023), data presented here establish that *rop* ^2xKO^ mutants also exhibit WT levels of the endogenous auxin IAA, normal distribution of the auxin transporter PpPINA, and unaltered expression of auxin‐regulated genes (Fig. S7). These findings establish that PpROP activity, like PpRIC, inhibits caulonema differentiation without affecting auxin‐controlled gene expression, further supporting the notion that PpROP activity and PpRIC function together in the same pathway to suppress caulonema differentiation.

Consistent with the substantially higher growth rate of caulonemal than chloronemal filaments (Cove & Knight, 1993), enhanced caulonema differentiation markedly increased the size of 5‐wk‐old *ric‐1* ^KO^ colonies (Ntefidou *et al*., 2023). By contrast, despite similarly enhanced caulonema differentiation, the size of *rop* ^2xKO^ colonies was significantly reduced at the same developmental stage (Fig. 1e,f). These findings suggest that PpRIC is specifically required to inhibit caulonema differentiation, whereas PpROP activity, as previously reported (Burkart *et al*., 2015; Cheng *et al*., 2020; Yi & Goshima, 2020; Bao *et al*., 2022), also promotes cell and protonemata expansion, evidently through the stimulation of other effectors. Importantly, both *ric‐1* ^KO^ and *rop* ^2xKO^ mutants only displayed minor growth defects, which were barely detectable and did not interfere with the demonstration of enhanced caulonema differentiation in 5‐d‐old protonemata, but became clearly apparent in 5‐wk‐old colonies.

Upon overexpression, PpROP1 (*c*. 30× increase in total Pp*ROP* gene expression as compared to WT; Fig. 6a) not only depolarized tip growth as discussed above (Fig. 6c–e) but also completely blocked caulonema differentiation (Fig. 6b), which together resulted in a strongly reduced colony size (Fig. 6f–h). By contrast, PpRIC overexpression (*c*. 40× WT Pp*RIC* expression level) also strongly inhibited caulonema differentiation but did not affect tip growth and, consequently, comparably mildly reduced colony size (Ntefidou *et al*., 2023). Together, these findings further support the conclusion that PpROP activity, as proposed above, inhibits caulonema differentiation via PpRIC activation and interacts with other effectors to promote directional cell expansion.

As indicated in the Results section, caulonema differentiation is tightly correlated with enhanced cell expansion, indicating interdependence between these processes (Jang & Dolan, 2011). Whereas PpRIC overexpression directly blocks caulonema differentiation without affecting cell expansion (Ntefidou *et al*., 2023), PpROP1 overexpression appears to inhibit caulonema differentiation not only directly via PpRIC stimulation, but also indirectly by disrupting tip growth. Similarly, the inhibition of caulonema differentiation observed at PpROP activity levels lower than those displayed by *rop* ^2xKO^ mutants seems more likely to be a consequence of severely reduced directional cell expansion (Fig. 9) than to indicate a direct role of low‐level PpROP activity in the stimulation of caulonema differentiation.

### PpROP activity contributes to the maintenance of apical initial cell identity

Protonemal filaments elongate based on tip growth and regular division of apical initial cells, which undergo gradual caulonema differentiation. PpROP activity appears to play an important role in the maintenance of apical initial cell identity not only by stimulating tip growth and by inhibiting caulonema differentiation, as discussed above, but also by promoting the rate of cell division. Supporting this notion and consistent with previously reported functions of mammalian RHO GTPases in stimulating cell proliferation (Coleman *et al*., 2004), cell division rates were increasingly reduced in *rop* ^3xKO^ and in *rop* ^4xKO^ mutants (Fig. 9). Rapid cell expansion enhances the proliferation of fission yeast and meristematic Arabidopsis cells (Fantes & Nurse, 1977; Jones *et al*., 2017). Consistent with these observations, caulonema differentiation induces *P. patens* protonemal cells not only to grow faster but also to divide more frequently (Jang & Dolan, 2011). PpROP activity may therefore promote the division of these cells indirectly by increasing the rate of tip growth, rather than directly by accelerating cell cycle progression. Alternatively, PpROP activity may stimulate both these processes.

### PpROP activity induces the formation of gametophore‐like structures and promotes the growth of regular gametophores

Regular gametophore formation was entirely abolished by high‐level PpROP overexpression, as well as at levels of total PpROP activity lower than those displayed by *rop* ^3xKO^ mutants (Fig. 9). The same conditions also completely blocked caulonema differentiation (Fig. 9), which was proposed to be essential for regular gametophore formation (Cove & Knight, 1993; Schumaker & Dietrich, 1997; Brun *et al*., 2003; Harrison *et al*., 2009). Strongly enhanced or reduced PpROP activity therefore appears to indirectly prevent regular gametophore formation by disrupting caulonema differentiation. Consistent with this conclusion, inhibiting caulonema differentiation by PpRIC overexpression also completely blocked gametophore formation (Ntefidou *et al*., 2023).

However, in liquid culture medium, estradiol‐induced PpROP2 expression at low levels, insufficient for regular gametophore formation, or PpROP1^Q64L^ expression at endogenous levels, induced and supported the development of gametophore‐like structures composed of rudimentary phyllids without rhizoids, which were never formed in the complete absence of PpROP activity (Fig. 9). Whereas regular gametophores develop from caulonema cells undergoing a well‐defined series of asymmetric and symmetric cell divisions (Moody *et al*., 2018), gametophore‐like structures emerged from undifferentiated or chloronemal cells dividing in a comparably random fashion (Figs 9, S9). Despite these differences, the observation that low‐level PpROP activity can induce the formation of aberrant gametophore‐like structures suggests that, at higher levels enabling caulonema differentiation, this activity may also play an important role in initiating regular gametophore development. Furthermore, regular gametophore growth clearly depends on PpROP activity, since *rop* ^2xKO^ *and rop* ^3xKO^ mutants, as well as complemented *rop* ^4xKO^ mutants displaying similar levels of total PpROP activity, formed gametophores with small leafy shoots and stunted rhizoids (Fig. 9). These observations demonstrate that PpROP activity not only has essential functions in promoting tip growth responsible for the elongation of protonemal filaments and rhizoids but also plays an important role in stimulating other forms of directional cell expansion, which are required for the growth of leafy shoots.

### GDP/GTP cycling is essential for PpROP functions in fundamental developmental processes

When expressed at the endogenous level in *rop* ^4xKO^ mutants, both WT PpROPs (Fig. 3) and fast‐cycling PpROP1^F31L^ (Fig. 7) essentially restored the *rop* ^3xKO^ phenotype. The F31L substitution in PpROP1 mirrors mutations, such as F28L in H‐RAS^F28L^ and analogous changes in mammalian RHO GTPases, which were biochemically confirmed to induce fast‐cycling behavior. Phenylalanine (F) at position 28 in H‐RAS and position 31 in PpROP1, along with other residues that directly interact with the guanine base of bound nucleotides (Reinstein *et al*., 1991), are located within essential domains required for GDP/GTP binding and are almost invariably conserved across RAS, RHO, and ROP family members. PpROP1^F31L^ is therefore expected to exhibit fast‐cycling behavior, although this remains to be confirmed by further investigation of this mutant and of analogous variants of other plant ROPs.

In contrast to fast‐cycling ROP variants, constitutively active GTP‐locked ROP mutants analogous to H‐RAS^Q61L^ or H‐RAS^G15V^, which are defective in GTP hydrolysis, have been extensively characterized in a variety of plant systems (e.g. Kost *et al*., 1999; Li *et al*., 1999; Zheng *et al*., 2002; Klahre & Kost, 2006). Unlike PpROP1^F31L^, at endogenous expression level, GTP‐locked PpROP1^Q64L^ was unable to complement defects in cell polarization and parallel cell plate positioning displayed by *rop* ^4xKO^ mutants, but it induced the formation of gametophore‐like structures by these mutants in liquid medium (Fig. 9). These observations demonstrate that GDP/GTP cycling is essential for PpROP functions in fundamental developmental processes underlying filament formation, but it is not necessary for the induction of gametophore‐like structures or directional cell expansion responsible for the growth of these structures. Further investigations are required to determine whether GDP/GTP cycling is also essential for other PpROP functions, such as the promotion of tip growth, which requires cell polarization, or the inhibition of caulonema differentiation, which depends on the formation of elongating filaments. While RHO GTPase functions generally appear to depend on GDP/GTP cycling, atypical endogenous RHO GTPases have been identified in animals, which, like PpROP1^Q64L^, can regulate specific cellular or developmental processes in a constitutively GTP‐bound state (Hodge & Ridley, 2016; Aspenström, 2022).

### Evolutionary origin and maintenance of the Pp*ROP* gene family

*Physcomitrium patens* protonemata express at similar levels four Pp*ROP* genes encoding nearly identical proteins, which, as demonstrated by data presented here, have essentially redundant functions and together provide total PpROP activity that governs key processes underlying protonemal development. Such a high degree of sequence conservation and functional integration is uncommon among eukaryotic gene families and raises important questions concerning the origin and maintenance of the Pp*ROP* gene family during evolution. While a comprehensive analysis of the underlying evolutionary mechanisms is beyond the scope of this study, several noteworthy observations are briefly discussed below, which provide relevant insights and a foundation for future in‐depth investigations.

At least one *ROP* gene is generally expressed in all plants (viridiplantae) with the exception of some green algae of the chlorophyte group (Ntefidou *et al*., 2023; Mulvey & Dolan, 2023a,b). *ROP* gene families typically have between four and > 10 members in ferns, gymnosperms, and flowering plants, but are much smaller with only one or two members in most plants displaying more ancient features, which include lycophytes, the simplest vascular plants, along with mosses and all other nonvascular plants (Mulvey & Dolan, 2023b). Interestingly, members of well‐characterized flowering plant *ROP* gene families cluster into four groups based on sequence similarity. Genes within each group generally appear to share similar expression patterns and functions, which are clearly distinct from those displayed by members of the other groups (Zheng & Yang, 2000; Christensen *et al*., 2003; Eklund *et al*., 2010a). Similarly, *RIC* gene families underwent substantial expansion, structural diversification, and functional differentiation with the emergence of flowering plants (Ntefidou *et al*., 2023). Altogether, these observations suggest that the evolution of structurally increasingly complex vascular plants was accompanied by the diversification of ROP functions, and that one or two ROP isoforms are generally sufficient to exert comparably simple functions in plants with more ancient features. Consequently, total PpROP activity regulating *P. patens* protonemal development, which is provided by four co‐expressed, nearly identical, and functionally redundant PpROP family members, appears to correspond to the activity of one or two ROP isoforms in other nonvascular plants and in lycophytes. Consistent with this conclusion, all ROPs expressed in different moss species share at least 93.9% identical amino acids with PpROP1 (Table S7), suggesting that ROP functions are highly conserved in all mosses.

The four members of the Pp*ROP* family possibly originated from a single ancient ROP gene as a consequence of two rounds of whole‐genome duplications (WGDs), which have occurred during the evolution of the *P. patens* genome (Rensing *et al*., 2007; Lang *et al*., 2018; Leebens‐Mack *et al*., 2019; Gao *et al*., 2022). Supporting this notion, the four Pp*ROP* loci are located on different chromosomes in regions which all appear to originate from ancestral chromosome I, one of seven chromosomes predicted to have existed before the two WGDs (Lang *et al*., 2018; Bi *et al*., 2024). While available data strongly support this ancestry for Pp*ROP1*, Pp*ROP3*, and Pp*ROP4*, the Pp*ROP2* locus is situated at the imprecisely defined junction between fused fragments derived from ancestral chromosomes I and II (Fig. S10). Interestingly, two consecutive WGDs and *ROP* gene families with four members have also been identified in mosses of the genus *Sphagnum*, whereas more than one WGD seems to have rarely occurred during the evolution of nonvascular plants with smaller *ROP* gene families (Table S7; Lang *et al*., 2018; Leebens‐Mack *et al*., 2019; Gao *et al*., 2022; Mulvey & Dolan, 2023a; Mulvey & Dolan, 2023b).

Remarkably, the four Pp*ROP* genes, which encode nearly identical proteins and possibly originate from genome duplications, were maintained in *P. patens* evidently in the absence of selective pressure driving their functional diversification. Although some degree of functional redundancy commonly appears to be evolutionarily stable, particularly in gene families with important signaling functions (Vieten *et al*., 2005; Cevik *et al*., 2023; Kumar & Ruiz, 2023), the selective advantage of this phenomenon is not fully understood (Dean *et al*., 2008) and is subject to ongoing further investigation (Nowak *et al*., 1997; Raval *et al*., 2023). Genomic functional redundancy can provide resilience against loss‐of‐function mutations (Z. Gu *et al*., 2003), as exemplified by the essentially normal development of *P. patens rop* ^1xKO^ mutants; and increases gene dosage, which was proposed to enhance plant adaptability to changing environmental conditions (Kondrashov *et al*., 2002). Moreover, according to the gene balance hypothesis, following WGDs, redundant genes are preferentially retained if their products participate in essential dose‐sensitive interactions. In such cases, the loss of a single gene copy may disrupt the stoichiometric balance of critical protein complexes or pathways, potentially compromising viability (Birchler & Veitia, 2010). It appears plausible that essential signaling functions of PpROPs may depend on dose‐sensitive interactions with other proteins. The retention of redundant genes may also be promoted by a reduction in their cumulative expression to a level matching the essential expression threshold of an ancestral progenitor gene (Qian *et al*., 2010). Consistent with a possible role of this mechanism in the evolutionary maintenance of the Pp*ROP* gene family, stepwise reductions in total Pp*ROP* expression resulting from sequential knockout of increasing numbers of family members progressively enhanced the severity of observed phenotypes.

### Outlook

Evolutionarily conserved essential functions of ROP GTPases in cell polarization, directional cell expansion, and cell plate positioning have previously been established based on the investigation of defects caused by loss‐of‐function or gain‐of‐function mutations in the moss *P. patens* (Burkart *et al*., 2015; Cheng *et al*., 2020; Yi & Goshima, 2020; Bao *et al*., 2022), in the nonvascular liverwort *M. polymorpha* (Mulvey & Dolan, 2023a), as well as in the flowering plants *A. thaliana* (Roszak *et al*., 2021) and *Zea mays* (Humphries *et al*., 2011). Data shown here demonstrate that during *P. patens* protonemal development, PpROP activity has additional important functions in inhibiting caulonema differentiation, in controlling gametophore development, and in the direct or indirect regulation of cell proliferation. Whether ROP GTPases have similar or related functions also in other plants represents an important area of future research.

The discoveries summarized in the previous paragraph have provided an increasingly detailed understanding of ROP functions in the control of different cellular and developmental processes in plants. However, the signaling network stimulated by ROP activity to regulate these processes has not been well characterized. Compared with ROP and RHO GTPase functions, this network appears to be much less evolutionarily conserved. In line with this notion, data presented here demonstrate that AtROP7 and HsRHOA can only complement the *rop* ^4xKO^ phenotype to a very limited extent. Although these heterologous RHO GTPases are closely related to PpROPs (85.2–85.7% (AtROP7) and 49.7% (HsRHOA) amino acid identity), even upon massive overexpression, they are only able to restore developmental processes in *rop* ^4xKO^ mutants, which require minimal PpROP activity. Similarly, in *P. patens* knockout mutants lacking geranylgeranyltransferase type I activity, which, as discussed in the Results section, appear to be defective in PpROP signaling and display a *rop* ^4xKO^ phenotype, expression of a YFP‐AtROP1 fusion protein mutated to enable prenylation by farnesyltransferases restored cell polarization and parallel cell division plane positioning, but not tip growth or other developmental processes. Interestingly, when tested using the same approach, human HsRAC1 or HsKRAS4b did not show any ability to complement the loss of PpROP activity (Bao *et al*., 2022).

Consistent with the findings discussed in the previous paragraph, homologs of key animal RHO downstream effectors, such as PAKs or ROCKs, seem to be generally missing in plants. Furthermore, plant‐specific ROP effectors, such as RICs, ICR/RIPs, and RISAPs, which control directional cell expansion in flowering plants (Lavy *et al*., 2007; Yalovsky *et al*., 2008; Stephan *et al*., 2014), appear to be missing or have different functions in *P. patens*. The *P. patens* genome encodes no clearly identifiable ICR/RIP or RISAP homologs and only a single member of the RIC family (Eklund *et al*., 2010a; Ntefidou *et al*., 2023), which is structurally very different from all its 11 *A. thaliana* homologs and does not regulate directional cell expansion, but inhibits caulonema differentiation (Ntefidou *et al*., 2023). Consequently, nothing is currently known about the effectors regulating tip growth downstream of PpROP activity in *P. patens*, and PpRIC is the only PpROP effector with a demonstrated function in the control of other cellular or developmental processes in this moss (Fig. 9). To further characterize the signaling network activated by PpROPs, it will be essential to identify and functionally characterize novel interaction partners and potential effectors of these proteins. The same approach will also provide important insights into the adaptation of RHO downstream signaling during evolution.

## Competing interests

None declared.

## Author contributions

ALB, MN and BK planned and designed the research. ALB, MN, JN, TIL, DK, AK, KL, HV and SS performed the experiments. ALB, BK, MN, AK and KL analyzed the data. BK and MN wrote the manuscript. BK and MN supervised the research. BK provided the project administration. KL and BK acquired the funding. ALB and BK contributed equally to this work.

## Disclaimer

The New Phytologist Foundation remains neutral with regard to jurisdictional claims in maps and in any institutional affiliations.

## Supporting information


**Fig. S1** Editing of the genomic Pp*ROP1* locus through homologous recombination and summary of transgenic lines.
**Fig. S2** Editing of the genomic Pp*ROP2* and Pp*ROP3* loci through homologous recombination.
**Fig. S3** Editing of the genomic Pp*ROP4* locus through homologous recombination.
**Fig. S4** Pp*ROP* gene expression in protonemata.
**Fig. S5** Knockout of a single Pp*rop* (*rop* ^1xKO^) does not affect protonemata.
**Fig. S6** Apical cells of *rop* ^2xKO^ and *rop* ^3xKO^ protonemata.
**Fig. S7** PpROPs do not influence the expression of auxin‐regulated genes or the auxin content.
**Fig. S8** β‐estradiol does not influence Pp*ROP* expression or protonemal development.
**Fig. S9** Gametophore‐like structures of *rop* ^4xKO^/*ROP1* ^pro^:*ROP1* ^Q64L^.
**Fig. S10** Genomic loci of Pp*ROP*s indicate a common origin from two WGD events.

**Table S1** *Physcomitrium patens* lines used in this study.
**Table S2** Culture media used in this study.
**Table S3** Oligonucleotides used in this study.
**Table S4** Vectors used in this study.
**Table S5** Statistical analyses.
**Table S6** Rate of polarized cell expansion of *rop* ^4xKO^/*ind* ^pro^:*ROP2* at low induction level.
**Table S7** ROP protein families in mosses: number of members and amino acid sequence conservation.
Please note: Wiley is not responsible for the content or functionality of any Supporting Information supplied by the authors. Any queries (other than missing material) should be directed to the *New Phytologist* Central Office.

## Data Availability

The data that support the findings of this study are available in the Supporting Information of this article (Fig. S1–S10; Tables S1–S7). Sequence data from this article can be found in the libraries of Phytozome *P. patens* V6.1 (Joint Genome Institute; Goodstein *et al*., 2012; Bi *et al*., 2024), TAIR (Arabidopsis Information Resource; Berardini *et al*., 2015), ONEKP cngbdb (Leebens‐Mack *et al*., 2019), and GenBank (Clark *et al*., 2016) under the following accession nos.: Aa*ROP* (onekp|ZTHV_scaffold_2084304 *Atrichum_angustatum*, CNGBdb); Aat*ROP1* (onekp|QMWB_scaffold_2004844 *Anomodon_attenuatus*, CNGBdb); Aat*ROP2* (onekp|QMWB_scaffold_2004846 *Anomodon_attenuatus*, CNGBdb); Aat*ROP3* (onekp|QMWB_scaffold_2004845 *Anomodon_attenuatus*, CNGBdb); Ar*ROP* (gnl|onekp|WOGB_scaffold_2094631 *Andreaea_rupestris*, CNGBdb); At*ROP7* (At5g45970, TAIR); Ba*ROP* (gnl|onekp|JMXW_scaffold_2006533 *Bryum_argenteum*, CNGBdb); Bap*ROP* (gnl|onekp|HRWG_scaffold_2070290 *Buxbaumia_aphylla*, CNGBdb); Cc*ROP* (gnl|onekp|TAVP_scaffold_2005151 *Calliergon_cordifolium*, CNGBdb); Cd*ROP1* (gnl|onekp|MIRS_scaffold_2008952 *Climacium_dendroides*, CNGBdb); Cd*ROP2* (gnl|onekp|MIRS_scaffold_2008953 *Climacium_dendroides*, CNGBdb); Cp*ROP1* (gnl|onekp|FFPD_scaffold_2008289 *Ceratodon_purpureus*, CNGBdb); Cp*ROP2* (gnl|onekp|FFPD_scaffold_2009252 *Ceratodon_purpureus*, CNGBdb); Df*ROP* (gnl|onekp|AWOI_scaffold_2072508 *Diphyscium_foliosum*, CNGBdb); Fa*ROP* (gnl|onekp|DHWX_scaffold_2074728 *Fontinalis_antipyretica*, CNGBdb); Hs*RHOA* (NM_001664.4, GenBank); La*ROP* (gnl|onekp|VMXJ_scaffold_2010101 *Leucobryum_albidum*, CNGBdb); Ol*ROP1* (gnl|onekp|CMEQ_scaffold_2011516 *Orthotrichum_lyellii*, CNGBdb); Ol*ROP2* (gnl|onekp|CMEQ_scaffold_2013049 *Orthotrichum_lyellii*, CNGBdb); Pf*ROP* (gnl|onekp|ORKS_scaffold_2003761 *Philonotis_fontana*, CNGBdb); Pc*ROP* (onekp|SZYG_scaffold_2042265 *Polytrichum_commune*, CNGBdb); Pp*AUX1* (Pp6c16_1210, Phytozome); Pp*PINA* (Pp6c23_4450, Phytozome); Pp*ROP1* (Pp6c14_2140, Phytozome); Pp*ROP2* (Pp6c2_11900, Phytozome); Pp*ROP3* (Pp6c1_11060, Phytozome); Pp*ROP4* (Pp6c10_2660, Phytozome); Pp*RSL1* (Pp6c1_20350, Phytozome); Pp*SHI1* (Pp6c21_9000, Phytozome); and Pp*UBIQUITIN‐E2* (Pp6c12_2450, Phytozome); Rs*ROP1* (gnl|onekp|JADL_scaffold_2005567 *Rhynchostegium_serrulatum*, CNGBdb); Rs*ROP2* (*gnl|onekp|JADL_scaffold_2005566 Rhynchostegium_serrulatum*, CNGBdb); Sf*ROP1* (Sphfalx06G097600, Phytozome); Sf*ROP2* (Sphfalx13G088000, Phytozome); Sf*ROP3* (Sphfalx14G076800, Phytozome); Sf*ROP4* (Sphfalx18G051700, Phytozome); and Tl*ROP* (gnl|onekp|SKQD_scaffold_2079078 *Takakia_lepidozioides*, CNGBdb).
