# Supplementary material for: Essential developmental processes in Physcomitrium patens require distinct levels of total activity provided by functionally redundant PpROP GTPases
Source: New Phytol. 2025 Oct 5;248(6):2865–90. doi: 10.1111/nph.70603 (PMC12630451; doi:10.1111/nph.70603)
Supplement: Supplementary file 1 — Fig. S1 Editing of the genomic PpROP1 locus through homologous recombination and summary of transgenic lines. Fig. S2 Editing of the genomic PpROP2 and PpROP3 loci through homologous recombination. Fig. S3 Editing of the genomic PpROP4 locus through homologous recombination. Fig. S4 PpROP gene expression in protonemata. Fig. S5 Knockout of a single Pprop (rop 1xKO) does not affect protonemata. Fig. S6 Apical cells of rop 2xKO and rop 3xKO protonemata. Fig. S7 PpROPs do not influence the expression of auxin‐regulated genes or the auxin content. Fig. S8 β‐estradiol does not influence PpROP expression or protonemal development. Fig. S9 Gametophore‐like structures of rop 4xKO/ROP1 pro:ROP1 Q64L. Fig. S10 Genomic loci of PpROPs indicate a common origin from two WGD events. [file NPH-248-2865-s002.docx]

## *New Phytologist* Supporting Information

**Article title:**

**Essential developmental processes in *P. patens* require distinct levels of total activity provided by functionally redundant PpROP GTPases**

**Authors:** Aude Le Bail, Benedikt Kost, Janina Nüssel, Tamara Isabeau Lolis, David Koch, Hildegard Voll, Sylwia Schulmeister, Alexander Kaier, Karin Ljung, Maria Ntefidou

**Article acceptance date: 09 September 2025**

The following Supporting Information is available for this article:

**Fig. S1** Editing of the genomic Pp*ROP1* locus through homologous recombination and summary of transgenic lines.

**Fig. S2** Editing of the genomic Pp*ROP2* and Pp*ROP3* loci through homologous recombination.

**Fig. S3** Editing of the genomic Pp*ROP4* locus through homologous recombination.

**Fig. S4** Pp*ROP* gene expression in protonemata.

**Fig. S5** Knock-out of a single Pp*ROP* (*rop*^1xKO^) does not affect protonemata.

**Fig. S6** Apical cells of *rop*^2xKO^ and *rop*^3xKO^ protonemata.

**Fig. S7** PpROPs do not influence the expression of auxin-regulated genes or the auxin content.

**Fig. S8** β-estradiol does not influence Pp*ROP* expression or protonemal development.

**Fig. S9** Gametophore-like structures of *rop*^4xKO^/*ROP1*^pro^:*ROP1*^Q64L^.

**Fig. S10** Genomic loci of Pp*ROP*s indicate a common origin from two WGD events.

conservation.


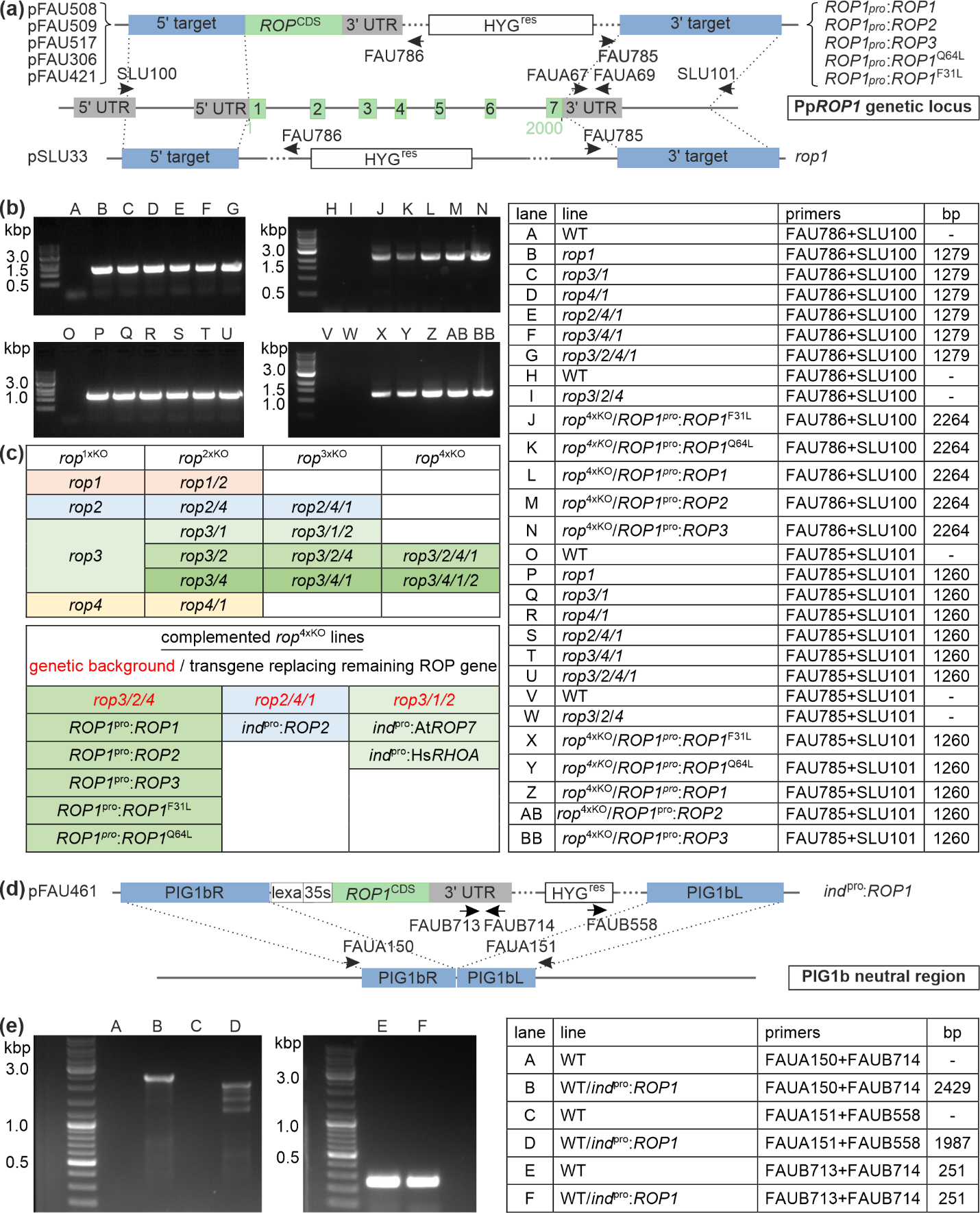


**Fig. S1 Editing of the genomic Pp*ROP1* locus through homologous recombination and summary of transgenic lines**. **(a)** Schematic representation of the Pp*ROP1* genetic locus and plasmid maps used to generate transgenic lines (Supporting Information Table **S1**), drawn to scale. pSLU33 was used to generate *rop1* knock-out by replacing the genomic fragment containing all exons and introns of Pp*ROP1* with the expression cassette, conferring resistance to hygromycin. pFAU508 (*ROP1^pro^*:*ROP1*), pFAU509 (*ROP1*^pro^:*ROP2*), pFAU517 (*ROP1*^pro^:*ROP3*), pFAU306 (*ROP1*^pro^:*ROP1*^Q64L^) or pFAU421 (*ROP1*^pro^:*ROP1*^F31L^) were used to generate *rop*^4xKO^ complementation lines by replacing all exons and introns of Pp*ROP1* in *rop3/2/4* with the coding and 3’ UTR sequence of a Pp*ROP* variant. **(a,d)** Green boxes: exons (numbered) or coding sequences, gray boxes: UTR sequences, blue boxes: regions used for homologous recombination targeting, white boxes: resistance markers, arrows: primers used for genotyping or RT-qPCR (Supporting Information Table **S3**), three dots: sequence not displayed to scale to save space, green numbers: nucleotide position in the Pp*ROP1* coding region. **(b)** Confirmation of transgenic lines through genotyping PCR using genomic DNA with the indicated primers. **(c)** Summary of *rop* knock-outs indicating the order in which they were generated from left to right (upper table). Summary of *rop*^4xKO^ complementation lines generated by replacement of the remaining Pp*ROP* in the indicated *rop*^3xKO^ genetic background (red font) with the coding sequence of an *ROP*/*RHO* gene expressed by the endogenous Pp*ROP1* promoter or the inducible β-estradiol-inducible promoter (Kubo *et al.*, 2013) (bottom table). **(d)** Vector map of pFAU461 used to overexpress *P*p*ROP1* in WT (WT/*ind*^pro^:*ROP1*) by inserting the coding and 3’ UTR sequence of Pp*ROP1* downstream of the inducible β-estradiol-inducible promoter in the PIG1 neutral region through homologous recombination. **(e)** Confirmation of WT/*ind*^pro^:*ROP1* transgenic line through genotyping PCR using genomic DNA with the indicated primers.


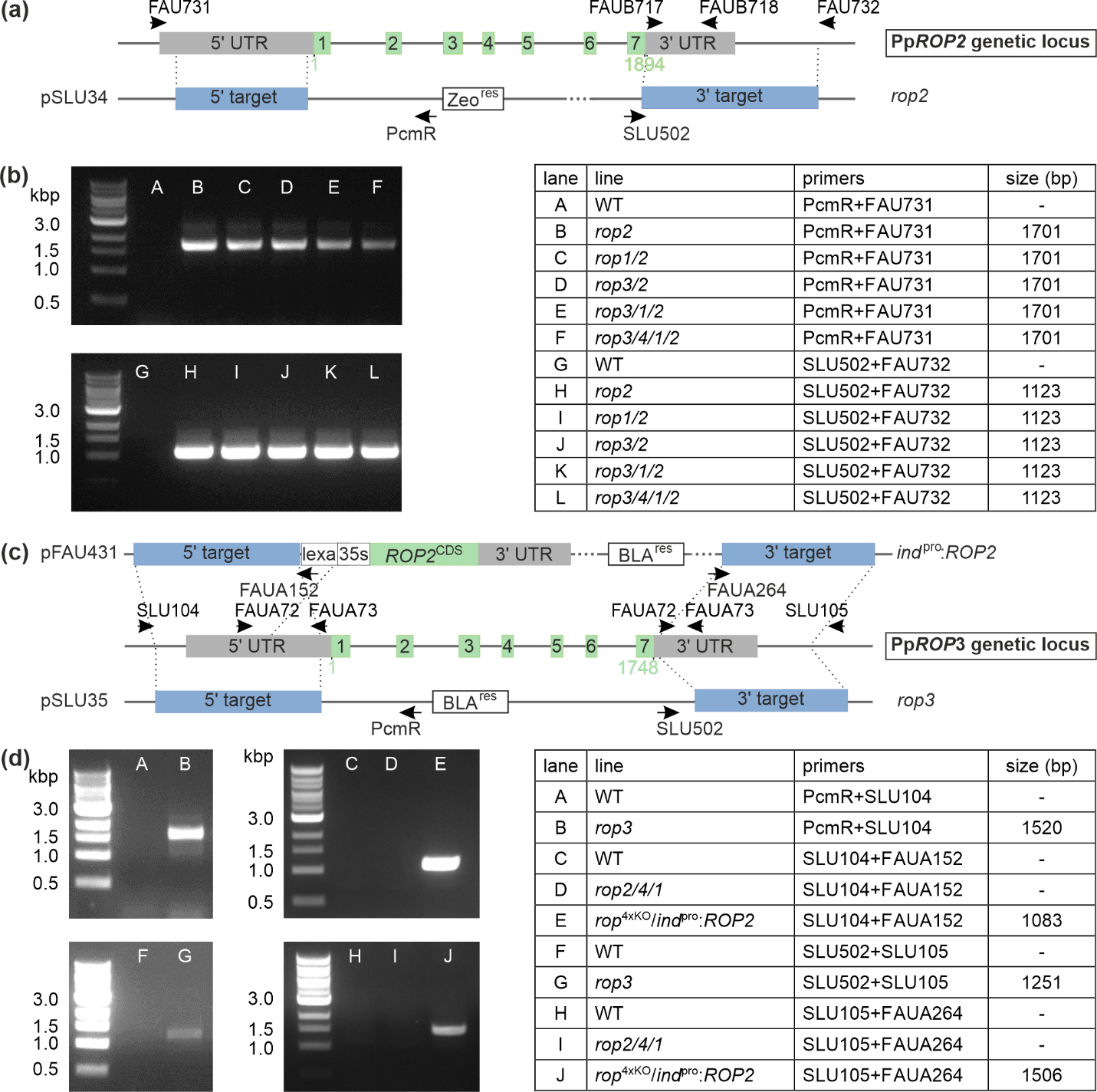


**Fig. S2 Editing of the genomic Pp*ROP2* and Pp*ROP3* loci through homologous recombination**. **(a,c)** Schematic representation of the Pp*ROP2* **(a)** and Pp*ROP3* **(c)** genetic loci and plasmid maps used to generate transgenic lines (Supporting Information Table **S1**), drawn to scale. Green boxes: exons (numbered) or coding sequences, gray boxes: UTR sequences, blue boxes: regions used for homologous recombination targeting, white boxes: resistance markers, arrows: primers used for genotyping or RT-qPCR (Supporting Information Table **S3**), three dots: sequence not displayed to scale to save space, green numbers: nucleotide position in the Pp*ROP2* **(a)** or Pp*ROP3* **(c)** coding sequence. **(a)** pSLU34 was used to generate *rop2* knock-out by replacing the genomic fragment containing all Pp*ROP2* exons and introns with the expression cassette, conferring resistance to zeocin. **(c)** pSLU35 was used to generate *rop3* knock-out by replacing the genomic fragment containing all Pp*ROP3* exons and introns with the expression cassette, conferring resistance to blasticidin. pFAU431 was used to complement *rop*^4xKO^ by replacing all exons and introns of the Pp*ROP3* locus with the coding and 3′ UTR sequence of Pp*ROP2* expressed under the control of the β-estradiol-inducible system (Kubo *et al.*, 2013) generating *rop*^4xKO^/*ind*^pro^:*ROP2*. The lexA operator and 35S promoter are depicted, but not the GX8 promoter or XVE regions. **(b,c)** Confirmation of transgenic lines through genotyping PCR using genomic DNA with the indicated primers.


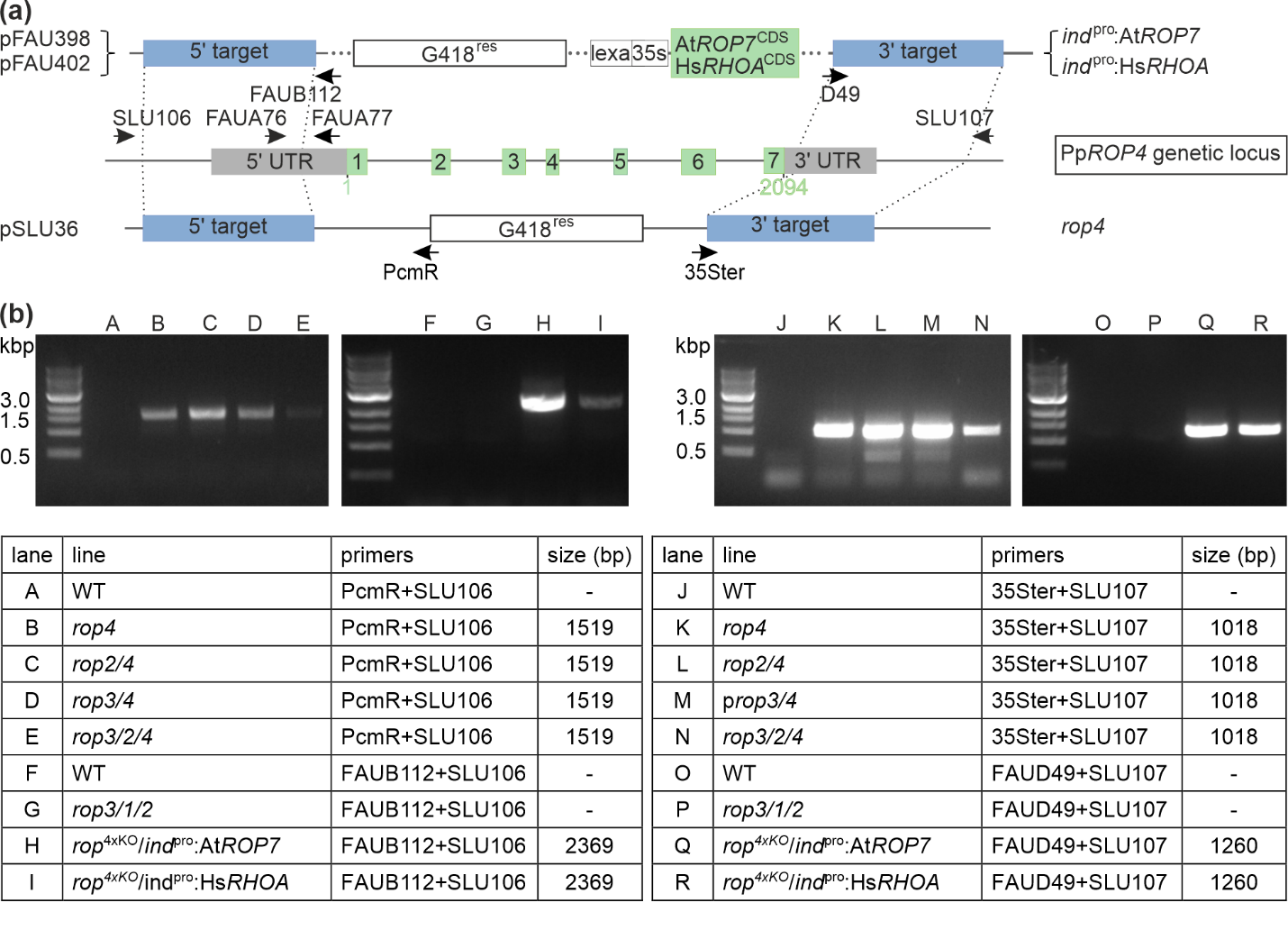


**Fig. S3 Editing of the genomic Pp*ROP4* locus through homologous recombination**. **(a)** Schematic representation of the Pp*ROP4* genetic locus and plasmid maps used to generate transgenic lines (Supporting Information Table **S1**), drawn to scale. pSLU36 was used to generate *rop4* knock-out by replacing the genomic fragment containing all Pp*ROP4* exons and introns with the expression cassette, conferring resistance to G418. pFAU398 (*ind*^pro^:At*ROP7*) or pFAU402 (*ind*^pro^*:*Hs*RHOA*) were used to complement *rop*^4xKO^ by replacing all exons and introns of Pp*ROP4* in *rop3/1/2* with the coding sequence of At*ROP7* or Hs*RHOA* expressed under the control of the β-estradiol-inducible system (Kubo *et al.*, 2013), generating *rop*^4xKO^/*ind*^pro^:At*ROP7* or *rop*^4xKO^/*ind*^pro^:Hs*RHOA*, respectively. Green boxes: exons (numbered) or coding sequences, gray boxes: UTR sequences, blue boxes: regions used for homologous recombination targeting, white boxes: resistance markers, arrows: primers (Supporting Information Table **S3**) used for genotyping or RT-qPCR, three dots: sequence not displayed to scale to save space, green numbers: nucleotide position in the Pp*ROP4* coding sequence. **(b)** Confirmation of transgenic lines through genotyping PCR using genomic DNA with the indicated primers.


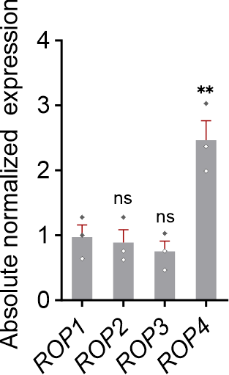


**Fig. S4 Pp*ROP* gene expression in protonemata**. Absolute transcripts levels of Pp*ROP*s in 1-week-old protonemata of WT cultivated through homogenization on BCDA medium (Supporting Information Table **S2**) were determined based on standard curves, using the value obtained for one WT replicate of Pp*ROP1* as calibrator (relative expression = 1). Bars represent means of three biological. Error bars: standard error of the mean (SEM). Statistical analysis by one-way ANOVA/Tukey’s test (Supporting Information Table **S5**): ^ns^ *P* > 0.05 (not significant); ** *P* ≤ 0.01.


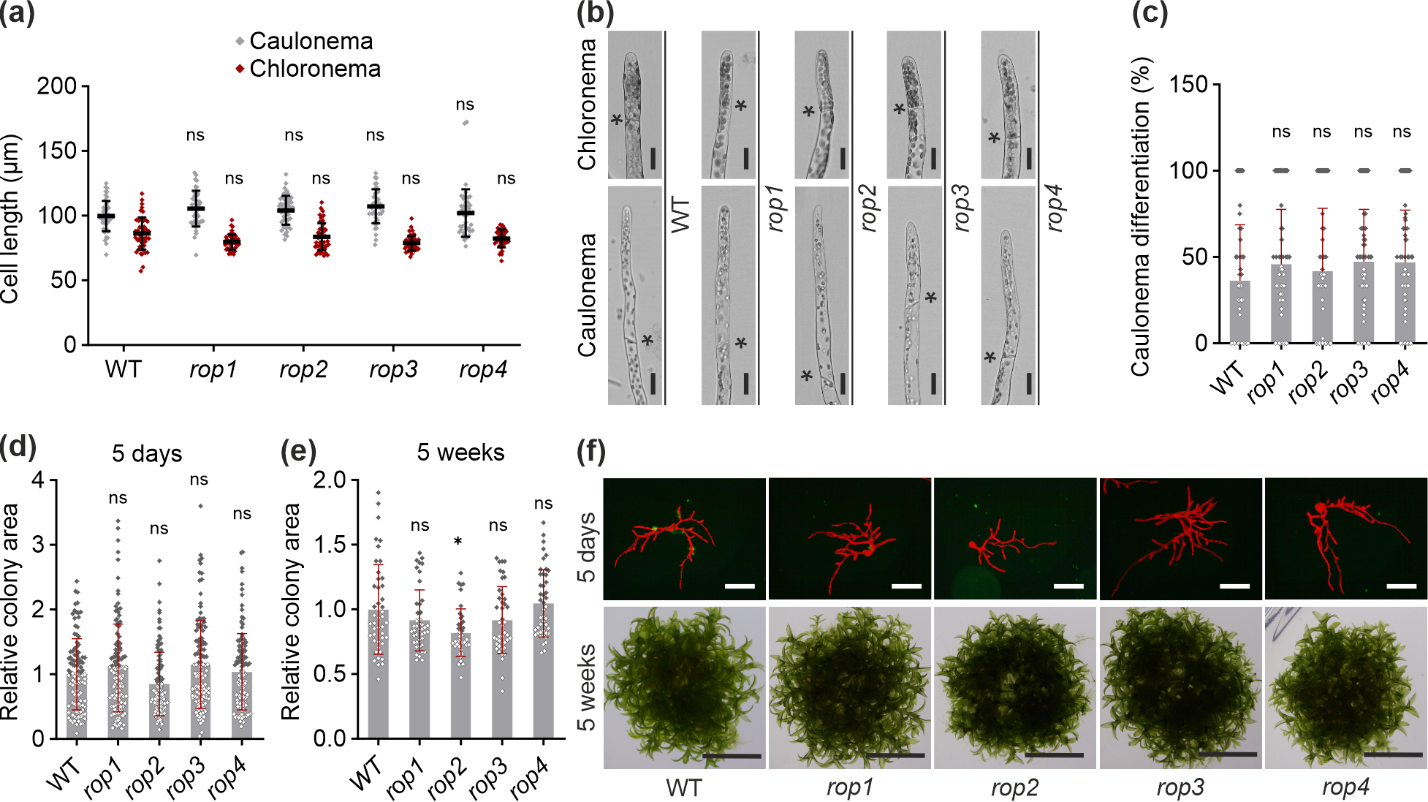


**Fig. S5** **Knock-out of a single Pp*ROP* (*rop*^1xKO^) does not affect protonemata**. **(a–f)** Graphs and images based on 5-day-old protonemata or 5-week-old colonies regenerated from protoplasts were cultivated using media listed in Supporting Information Table **S2**. **(a)** Average subapical cell length of chloronemata and caulonemata in 5-day-old protonemata. n = 50 cells per genotype. The experiment was repeated three times with consistent results. **(b)** Bright field micrographs of chloronemal and caulonemal filament tips. Asterisks: cell wall between apical and subapical cells. Scale bars: 25 µm. **(c)** Average percentage of caulonema differentiation in 5-day-old protonema filaments with at least three cells as determined by microscopic observation. n = 60 colonies per genotype measured in 3 independent experiments. **(d–f)** Average size of 5-day-old protonemata **(d)** or 5-week-old colonies **(e)** determined using micrographs of chlorophyll autofluorescence **(f**, upper row**)** or bright field images **(f**, lower row**)** recorded with a stereo microscope. n = 120 colonies per genotype measured in 3 independent experiments **(d)** or n = 41 colonies per genotype. The experiment was performed three times with consistent results **(e)**. Scale bars: 400 µm **(f,** upper row**),** 10 mm **(f,** lower row**)**. (**a, c–e)** Error bars: standard deviation (SD), dots represent individual data points. Statistical analysis by two-way ANOVA/Tukey’s test **(a)** or one-way ANOVA/Tukey’s test **(c–e)**. Pairwise comparisons to WT are displayed, all others see Supporting Information Table **S5**: ^ns^ *P* > 0.05 (not significant); * *P* ≤ 0.05.


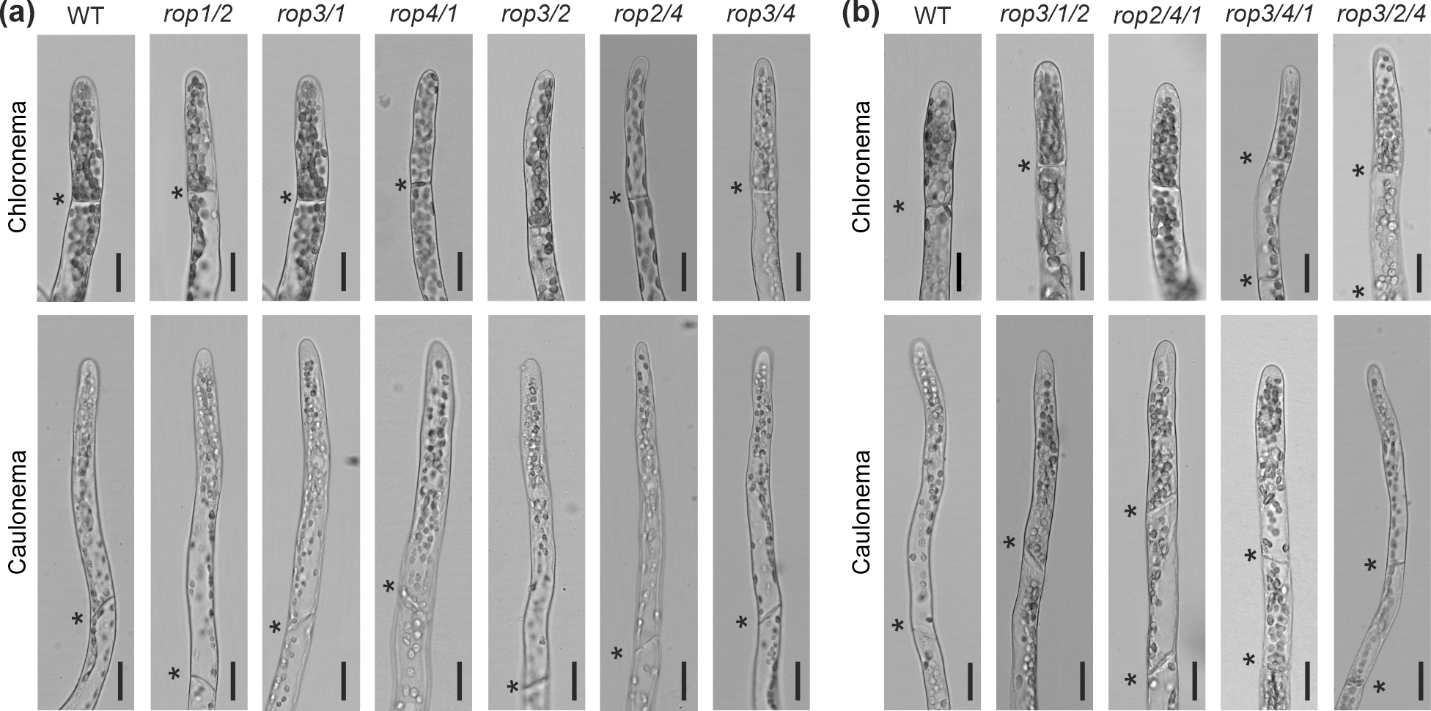


**Fig. S6 Apical cells of *rop*^2xKO^ and *rop*^3xKO^ protonemata.** Bright field images of 5-day-old chloronemal and caulonemal filament tips of *rop*^2xKO^ (a) and *rop*^3xKO^ (b) compared to WT. Asterisks indicate the cell wall between neighboring cells. Scale bars: 25 µm.


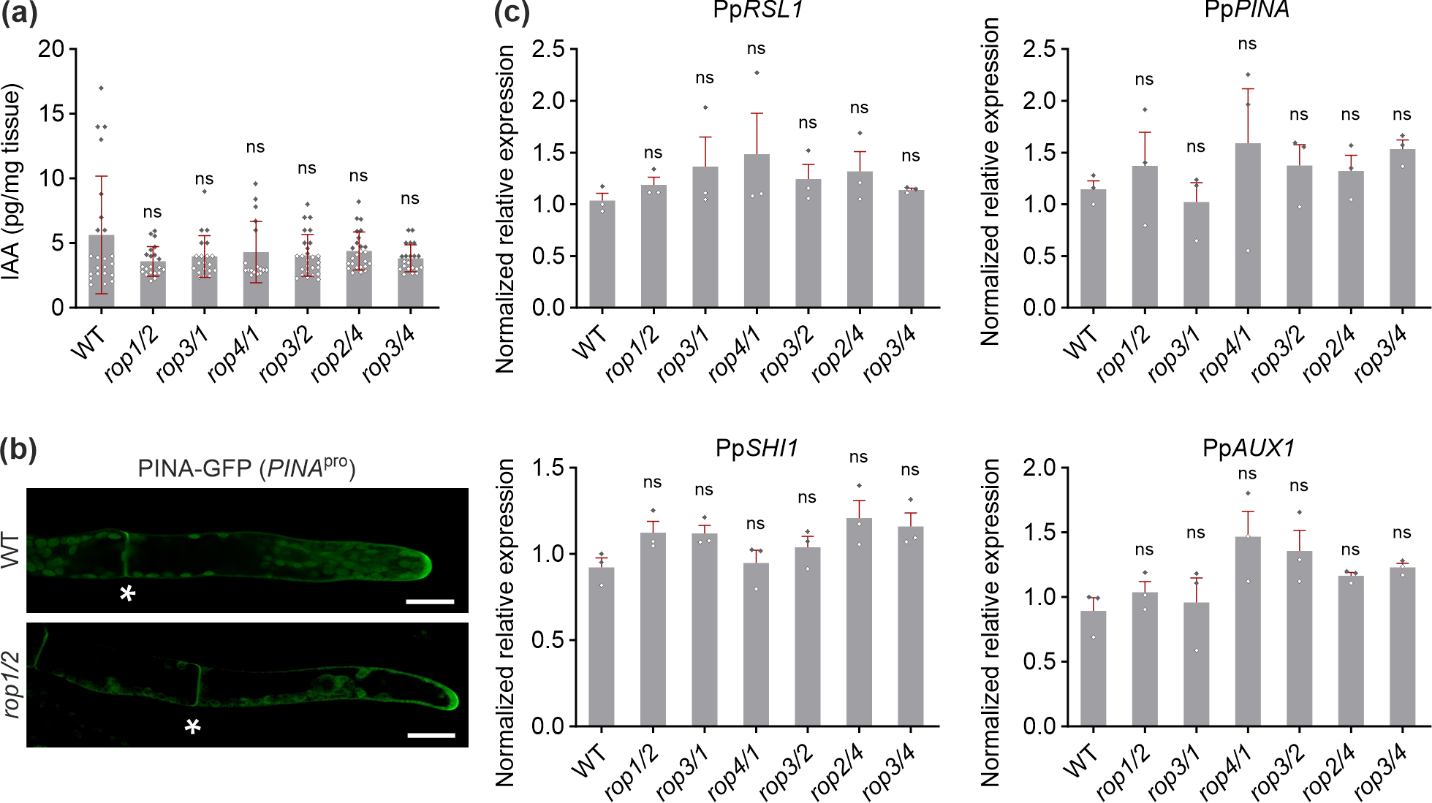


**Fig. S7 PpROPs do not influence the expression of auxin-regulated genes or the auxin content**. **(a–c)** Graphs and images based on 1-week-old protonemata with the indicated genotype cultivated using media described in Supporting Information Table **S2**. **(a)** Free IAA content of WT and *rop*^2xKO^ lines were analyzed by gas chromatography-tandem mass spectrometry. Bars: means of n = 11 independent measurements, error bars: standard deviation, dots represent individual data points. **(b)** Confocal microscopy imaging of PpPINA-GFP (Pp*PINA^pro^*:Pp*PINA*-*GFP*) (Viaene et al., 2014) in WT and *rop1*/*2*. Asterisks indicate the cell wall between the apical and subapical cell. Scale bars: 25 µm. **(c)** RT-qPCR was used to assess the expression of several auxin-responsive genes related to auxin signaling according to the 2^−ΔCCT^ method, using the value obtained for one WT replicate as a calibrator (relative expression = 1). Bars: mean of three biological replicates, error bars: standard error of the mean (SEM). **(a,c)** Statistical analysis by one-way ANOVA/Tukey’s test (pairwise comparisons to WT are displayed, all others see Supporting Information Table **S5**): ^ns^ *P* > 0.05 (not significant).


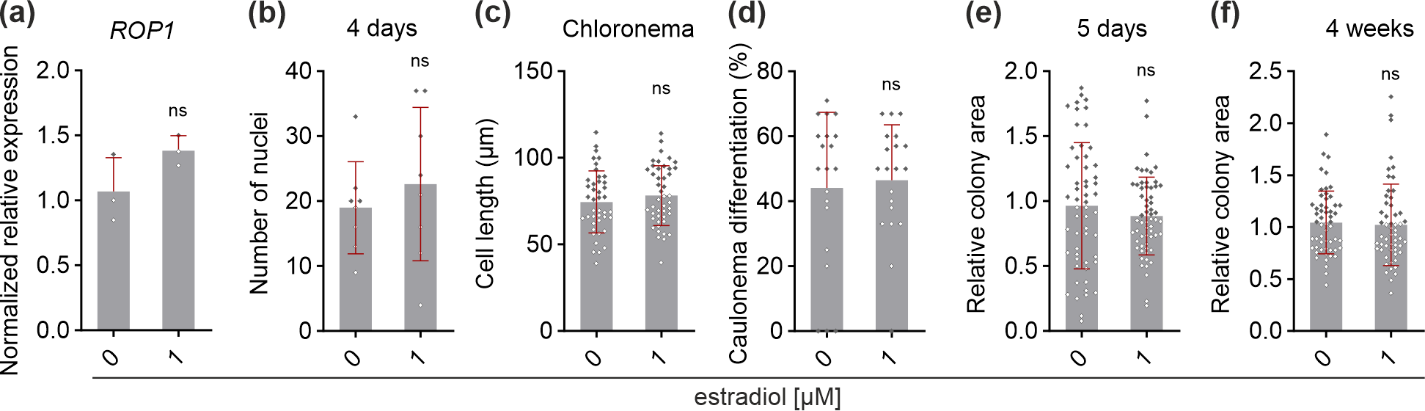


**Fig. S8 β-estradiol does not influence Pp*ROP* expression or protonemal development**. Graphs based on protonemata or 4-week-old colonies of WT cultivated using media listed in Supporting Information Table **S2**. 1 µM β-estradiol was added to the media from a 10 mM stock solution dissolved in DMSO. **(a)** Relative expression level of Pp*ROP1* in 1-week-old protonemata cultivated through homogenization was determined according to the 2^−ΔCCT^ method, using the value obtained for one WT replicate as calibrator (relative expression = 1). Bars: mean of three biological replicates; error bars: standard error of the mean (SEM). The experiment was repeated two times with consistent results. **(b–f)** Nuclei count and growth parameters were assessed using protoplasts regenerated for 2 days on PRMB medium without β-estradiol, followed by 2 days **(b)** or 3 days **(c–e)** on BCDA medium or 4 weeks **(f)** on BCD medium supplemented with 1 µM β-estradiol. **(b)** Nuclei were counted in 4-day-old protonemata stained with DAPI using confocal fluorescence microscopy. n = 8 colonies per genotype. The experiment was repeated three times with consistent results. **(c,d)** Average subapical cell length of chloronema cells **(c)** or average percentage of caulonema differentiation in 5-day-old protonemal filaments with at least three cells as determined by microscopic observation **(d)**. n = 42 cells per genotype were analyzed in 3 independent experiments **(c),** or n = 20 colonies per genotype were analyzed. The experiment was repeated three times with consistent results **(d)**. (**e,f)** Average size (area) of 5-day-old **(e)** or 4-week-old **(f)** colonies determined based on microscopic imaging of chlorophyll autofluorescence **(e)** or bright field micrographs **(f)** using a stereo microscope. The mean value of WT was used as a calibrator (relative area = 1). n = 55 protonemata **(f)** or n = 60 colonies **(e)** per genotype were analyzed in 3 independent experiments. (**a–f**) Error bars: standard error of the mean (SEM) **(a),** standard deviation (SD) **(c–f)**; dots represent individual data points. Statistical analysis (Supporting Information Table **S5**) by unpaired Student’s *t*-test: ^ns^ *P* > 0.05 (not significant).


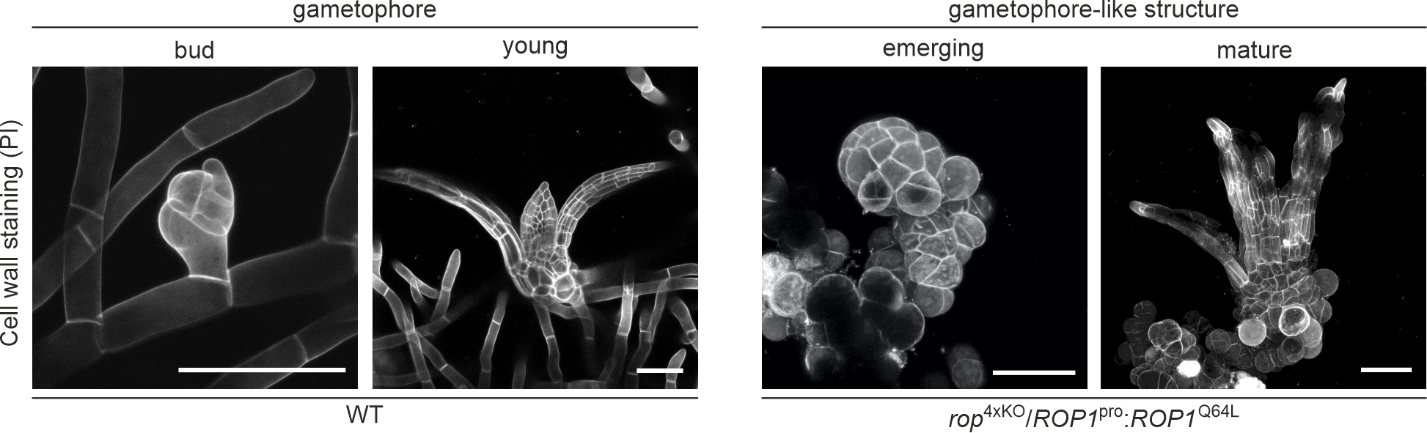


**Fig. S9 Gametophore-like structures of *rop*^4xKO^/*ROP1*^pro^:*ROP1*^Q64L^**. 2-week-old (WT) or 5-week-old (*rop*^4xKO^/*ROP1*^pro^:*ROP1^Q64L^)* were cultivated in liquid BCD medium (Supporting Information Table **S2**). Maximum projections of serial optical sections of cell walls stained with propidium iodide were imaged using confocal microscopy. Scale bars: 100 µm.


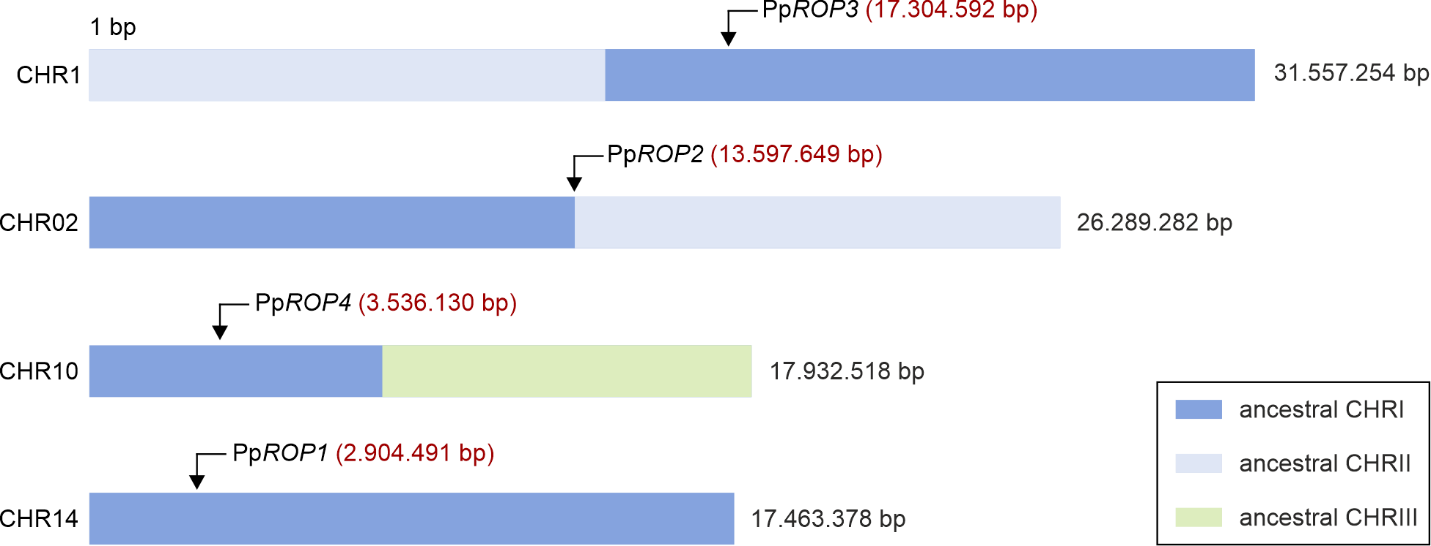


**Fig. S10 Genomic loci of Pp*ROP*s indicate a common origin from two WGD events**. Schematic representation of chromosomes 1, 2, 10 and 14, color-coded according to their origin from ancestral chromosomes I, II and III, based on the genome assembly v6.1 (Lang *et al.*, 2018; Bi *et al.*, 2024). Arrows: Pp*ROP* loci. Numbers (black font): first and last bp of chromosomes, numbers (red font): first bp of the 5' UTR of genomic Pp*ROP* sequences.

**References**

**Bi G, Zhao S, Yao J, Wang H, Zhao M, Sun Y, Hou X, Haas FB, Varshney D, Prigge M, et al. 2024.** Near telomere-to-telomere genome of the model plant Physcomitrium patens. *Nat. Plants* **10**: 327-343.

**Lang D, Ullrich KK, Murat F, Fuchs J, Jenkins J, Haas FB, Piednoel M, Gundlach H, Van Bel M, Meyberg R, et al. 2018.** The *Physcomitrella patens* chromosome-scale assembly reveals moss genome structure and evolution. *Plant J.* **93**(3): 515-533.

**Kubo M, Imai A, Nishiyama T, Ishikawa M, Sato Y, Kurata T, Hiwatashi Y, Reski R, Hasebe M. 2013.** System for Stable β-Estradiol-Inducible Gene Expression in the Moss Physcomitrella patens. PLoS One 8(9): e77356.

**Viaene T, Landberg K, Thelander M, Medvecka E, Pederson E, Feraru E, Cooper Endymion D, Karimi M, Delwiche Charles F, Ljung K, et al. 2014.** Directional auxin transport mechanisms in early diverging land plants. *Curr. Biol.* **24**(23): 2786-2791.
